# The effect of sexually transmitted co-infections on HIV viral load amongst individuals on antiretroviral therapy: a systematic review and meta-analysis

**DOI:** 10.1186/s12879-015-0961-5

**Published:** 2015-06-30

**Authors:** David Champredon, Steven E Bellan, Wim Delva, Spencer Hunt, Chyun-Fung Shi, Marek Smieja, Jonathan Dushoff

**Affiliations:** McMaster University, Hamilton, ON Canada; The University of Texas at Austin, Center for Computational Biology and Bioinformatics, Austin, TX USA; The South African DST/NRF Centre of Excellence in Epidemiological Modelling and Analysis (SACEMA), University of Stellenbosch, Stellenbosch, South Africa; International Centre for Reproductive Health (ICRH), Ghent University, Ghent, Belgium

## Abstract

**Background:**

Antiretroviral therapy (ART) markedly reduces HIV transmission, and testing and treatment programs have been advocated as a method for decreasing transmission at the population level. Little is known, however, about the extent to which sexually transmitted infections (STIs), which increase the HIV infectiousness of untreated individuals, may decrease the effectiveness of treatment as prevention.

**Methods:**

We searched major bibliographic databases to August 12^th^, 2014 and identified studies reporting differences in HIV transmission rate or in viral load between individuals on ART who either were or were not co-infected with another STI. We used hierarchical Bayesian models to estimate viral load differences between individuals with and without STI co-infections.

**Results:**

The search strategy retrieved 1630 unique citations of which 14 studies (reporting on 4607 HIV viral load measurements from 2835 unique individuals) met the inclusion criteria. We did not find any suitable studies that estimated transmission rates directly in both groups. Our meta-analysis of HIV viral load measurements among treated individuals did not find a statistically significant effect of STI co-infection; viral loads were, on average, 0.11 log10 (95 % CI −0.62 to 0.83) higher among co-infected versus non-co-infected individuals.

**Conclusions:**

Direct evidence about the effects of STI co-infection on transmission from individuals on ART is very limited. Available data suggests that the average effect of STI co-infection on HIV viral load in individuals on ART is less than 1 log10 difference, and thus unlikely to decrease the effectiveness of treatment as prevention. However, there is not enough data to rule out the possibility that particular STIs pose a larger threat.

**Electronic supplementary material:**

The online version of this article (doi:10.1186/s12879-015-0961-5) contains supplementary material, which is available to authorized users.

## Background

A large body of evidence suggests that antiretroviral therapy (ART), particularly with newer treatment regimens, markedly reduces the risk of sexual transmission of HIV. Recent systematic reviews have estimated that ART causes a more than ten-fold reduction in the incidence rate within discordant couples, to less than 0.5 per 100 person-years [1–6].

These sharp reductions have inspired the idea of antiretroviral treatment as prevention—aggressive programs to identify and treat HIV-positive individuals could substantially reduce HIV incidence at the population level, by reducing the infectiousness of HIV-infected individuals [5, 7]. However, increased infectiousness when treated individuals are co-infected with one or more other sexually transmitted infections (STIs) could potentially undercut the effectiveness of treatment as prevention programs. Concern with the effects of co-infection on HIV transmission is exemplified in the 2008 “Swiss Statement,” which argues that HIV sexual transmission risk is of no concern within stable discordant relationships in which: an HIV-positive partner is adhering to treatment under the care of a physician; the viral load has been suppressed for at least six months; and *no other STIs are infecting the HIV-positive partner* [8].

Although the biological mechanisms underlying this increased risk are not fully understood, many STIs have been associated with higher risks of both HIV acquisition and sexual transmission [9–15]. Increased HIV transmission may be underpinned by higher HIV viral load resulting from larger concentration of HIV-infected immune cells in genital secretions induced by an inflammatory response and/or additional pathways caused by genital ulcers [16]. Similarly, inflammatory STIs, by recruiting immune cells, may provide additional targets for HIV virions, increasing HIV acquisition risk. Ulcerative STIs may present additional entry points for HIV infection [15].

Studies of HIV-STI interactions have been conducted mostly on individuals *not* receiving ART. Less is known about the impact of STI co-infections on HIV shedding from treated individuals. STI prevalence is high among HIV-infected individuals [17] and the proportion of these individuals on ART is quickly rising [18]. Thus, any potential increased HIV infectiousness due to STI co-infections among treated individuals could have important epidemiological consequences as treatment as prevention becomes more widespread.

Disentangling the many interacting factors at play is challenging: many STIs are suspected of affecting HIV shedding [9] and it remains unclear whether how these effects interact in people with more than one such infection; the viral load response to ART is regimen- and gender-specific [19]; numerous (not necessarily consistent) methods are used to sample and quantify HIV viral load [20, 21]; viral load measurements can vary between anatomical sites within an infected individual [22]; and HIV viral loads exhibit substantial temporal variation [23]. When considering transmission events in discordant couples, isolating the effect of STI co-infections is challenging because concomitance of STI infections in both partners (that could affect both HIV susceptibility and infectiousness) and HIV transmission are often not practical to ascertain.

Here, we conduct a systematic review and meta-analysis of the available evidence to assess whether STI co-infections affect the risk of HIV transmission from individuals on ART. We searched for studies that estimated transmission directly, and also for studies that measured viral load, which we intended to use as a proxy for transmission if the direct evidence was insufficient.

## Methods

This systematic review and meta-analysis followed the guidelines from the PRISMA statement [24] (see Additional file 1). A protocol was prospectively registered in the PROSPERO database (see Additional file 2). Published peer-reviewed observational studies and randomized controlled trials were considered for inclusion. We included studies of sexually active HIV-infected participants on ART that were further classified into two subgroups: participants whose only known STI was HIV (the “mono-infected group”) and those with HIV and co-infected with another STI (the “co-infected group”). Studies were eligible for inclusion if they measured HIV viral loads among HIV-infected participants, or if they observed at least monthly HIV transmission events and STI infection status in discordant couples. An individual was considered co-infected only if the STI was laboratory confirmed. Individuals with ongoing treatment for the co-infecting STI were not included.

We searched for all relevant studies in Medline (Ovid), EMBASE (Ovid), PubMed, CINAHL and the Cochrane Library from inception to August 12^th^, 2014. Subject headings and text words associated with the risk of HIV sexual transmission, ART and STIs were included in the search strategies. We included STIs commonly discussed in the context of HIV transmission: *Chlamydia trachomatis*, chancroid (*H. ducreyi*), any type of Human Papilloma Virus, Herpes Simplex Virus 2, *Neisseria gonorrhoeae*, syphilis (*T. pallidum*) and Trichomoniasis (*T. vaginalis*). We also included bacterial vaginosis and candidal vaginitis, although these are not known to result directly from sexual transmission, and urethritis, which can be associated with more than one STI. The search did not impose any language or geographical restrictions on studies. The STI positivity definitions are given in Additional file 3 and full search strategies in Additional file 4.

All retrieved abstracts were read by three authors (DC, CS and SH). Eligibility assessment was performed independently by two authors for each abstract, using pre-defined guidelines. Disagreement between authors was resolved by consensus after discussion. Data from eligible studies were extracted independently by two authors (DC and SH).

We sought to assess how two primary outcomes of interest, HIV viral loads and HIV transmission rates within discordant couples, varied between HIV-infected participants on ART with and without STI co-infections. We sought additional data to assess potential sources of bias within and between studies including STI diagnostic methods; anatomical sites sampled for HIV viral load measurements; HIV assays; interval between STI co-infection diagnosis and HIV viral load measurement; ART regimen, treatment length and adherence; study design; HIV-infected participant age, gender, and sexual orientation; and, for serodiscordant couple studies, concomitance of STI co-infection in HIV-uninfected partner and HIV genetic linkage following secondary partner seroconversion. Within studies, we excluded individuals not explicitly known to be on ART and, when this information was available, those who had been on ART for less than 30 days. We probed study quality by summarizing variables and methods (Tables 1 and 2), and with forest and funnel plots (Figs. 3 and 4).

**Table 1:** Summary table of studies included in the meta-analysis

Study	Study objective	Country	Population	Coverage period	Study design	STI	STI assessment method	Number of participants	Individuals included in meta-analysis	HIV viral load measure	HIV VL anatimical site
Adolf 2012 [31]	Prevalence and risk factors for syphilis among HIV+	Brazil	Females and males; STI/HIV clinic patients-SoBrHIV cohort	1991–2008	Case-control	Tp	VDRL, FTA	1012	759	Not reported	Blood plasma
Anderson 2008 [32]	Association between presence of inflammatory	USA	Females; STI/HIV clinic patients	Not reported	Prospective cohort	Bv Ct Cv Ng Tp Tv	Bv: Amsel	97	41	RNA	Blood and CVL
							Ct, Ng: culture				
							Cv: visual exam				
							Tv: wet mount				
							Tp: RPR				
Conley 2010 [33]	Prevalence and risk factors for abnormal anal cytology among HIV+	USA	Females and males; STI/HIV clinic patients - SUN study	2004–2006	Prospective cohort	Ct HPV HSV Ng Tp Tv	HPV, Ng, Ct: NAAT, cytological	621	147	RNA	Not reported
							Tv: NAAT				
							HSV2: serology				
Chan 2008 [12]	Correlation between HIV VL in blood and semen among men both ART naive and experienced	Australia	Males; STI/HIV clinic patients	2003–2006	Prospective cohort	Ct Ng Tp	Ct: NAAT	119	81	RNA	Blood and semen
							Ng: culture				
							Tp: Fabs + RPR				
Cu-Uvin 2001 [26]	Impact of genital tract infections on HIV cervicovaginal shedding	USA	Females; STI/HIV clinic patients	Not reported	Prospective cohort	Bv Cv Tv	Bv: Amsel	108	61	RNA	Blood and CVL
							Cv, Tv: culture				
Graham 2011 [34]	Impact of genital ulcerations on HIV genital shedding	Kenya	Females; STI/HIV clinic patients	2004–2008	Prospective cohort nested	Bv Ct Cv Hd Ng Tp Tv	Tv: wet mount	145	37	RNA	Cervix and vagina
							Bv: Nugent				
							Cv: not reported				
							Ng: culture + NAAT				
							Ct: NAAT				
							Hd: culture				
							Tp: serology				
Jarzebowski 2012 [35]	Impact of syphilis on CD4 and HIV VL	France	Males; MSM-FHDH cohort	1998–2006	Case-control	Tp	Not reported	1515	1271	RNA	Blood plasma
Kofoed 2006 [36]	Impact of Syphilis infection on CD4, HIV VL and response after anti-treponemal treatment	Denmark	Males; MSM	2003–2004	Prospective experimental	Tp	Dark field, serology	38	34	RNA	Blood plasma
Madeddu 2014 [41]	HPV screening should be done even on HIV-positive women on ART	Italy	Females; STI/HIV clinic patients	2008–2009	Prospective cohort	HPV	NAAT	57	52	RNA	Blood plasma
Politch 2012 [37]	Prevalence of seminal HIV shedding among MSM on ART	USA	Males; MSM	Not reported	Prospective cohort	Ct HSV Ng Tp NGU	HSV: serology	101	96	DNA and RNA free and RNA assoc	Semen
							Ct, Ng, Tp: not reported				
Sadiq 2002 [38]	Effect of urethritis on seminal HIV VL for patients on ART	UK	Males; MSM	1998–2000	Prospective cohort	Ct Ng NGU	Ng: culture	40	39	RNA and DNA	Blood and semen
							Ct: NAAT				
Sha 2005 [39]	Association of Bv and Bv-associated bacteria with HIV genital VL	USA	Females; STI/HIV clinic patients	1994–1997	Prospective cohort	Bv Ct Cv HPV HSV Ng Tp Tv	Bv: Amsel + Nugent	362	107	RNA	Blood and CVL
							Tp: symptoms + DFA				
							Ct, Ng: culture + pap				
							HSV: symptoms + pap				
							HPV: NAAT				
Sudenga 2012 [40]	HSV2 epidemiology in HIV+/at risk adolescents	USA	Females and males; adolescent; REACH cohort	1996–2000	Case-control	Bv Ct Hd HPV HSV Ng Tp Tv	HSV2: serology	513	60	RNA	Blood plasma
							Ct, Ng, HPV: NAAT				
							Bv: gram stain + clinical criteria				
							Tv: culture				
Winter 1999 [10]	Impact of asymptomatic urtethritis on HIV VL in semen	UK	Males; STI/HIV clinic patients	Not reported	Prospective cohort	Ct Ng NGU	Ng: Gram stain, culture	94	53	RNA cell free	Blood and semen
							Ct: NAAT				

MSM: men who have sex with men

*Bv* Bacterial vaginosis, *Cv* Candidal vaginitis, *Ct Chlamydia trachomatis*, *HPV,* human papillomavirus, *HSV* human simplex virus type 2, *Ng Neisseria gonorrhoeae, Tp Treponema pallidum*, *Tv Trichomonas vaginalis*, *Ur* urethritis

**Table 2:** Assessment of risk of bias within studies

Study	Number of STIs tested	Sampling site for HIV	ART adherence reported
Adolf 2012 [31]	1	Blood plasma	No
Anderson 2008 [32]	6	Blood and CVL	No
Conley 2010 [33]	6	Not reported	No
Chan 2008 [12]	3	Blood and semen	No
Cu-Uvin 2001 [26]	3	Blood and CVL	No
Graham 2011 [34]	7	Cervix and vagina	Yes
Jarzebowski 2012 [35]	1	Blood plasma	No
Kofoed 2006 [36]	1	Blood plasma	No
Madeddu 2014 [41]	1	Blood plasma	Yes
Politch 2012 [37]	4	Semen	No
Sadiq 2002 [38]	2	Blood and semen	No
Sha 2005 [39]	8	Blood and CVL	No
Sudenga 2012 [40]	8	Blood plasma	Yes
Winter 1999 [10]	2	Blood and semen	No

Studies reporting a large number of STIs tested, *ART* adherence and measuring HIV, *VL* viral load in genital secretions are less likely to bias estimates of the effect of STI co-infection on HIV transmission, *CVL* cervicovaginal lavage

**Table 3:** Number of HIV viral loads measurements included in the meta-analysis by STI co-infection and anatomical sites

STI	Blood	Cervicovaginal	Semen	Total
Bv	51	52	n/a	103
Ct	9	0	9	18
Cv	2	9	n/a	11
HPV	260	76	0	336
HSV	86	0	60	146
Ng	9	2	9	20
Tp	656	2	2	660
Tv	4	4	0	8
Ur	9	n/a	12	21
none	2915	192	177	3284
Total	4001	337	269	4607

Category “none” means there was no STI (other than HIV) co-infecting the patient

*Bv* bacterial vaginosis, *Cv* candidal vaginitis, *Ct Chlamydia trachomatis*, *HPV* human papillomavirus, *HSV* human simplex virus type 2, *Ng Neisseria gonorrhoeae*, *Tp Treponema pallidum*, *Tv Trichomonas vaginalis*, *Ur* urethritis, *n/a* not applicable

Planned measures of effect were: the difference in log10 HIV viral load between the mono- and co-infected groups, for a given anatomical site and STI; and the relative HIV transmission rate between HIV-discordant couples with the infected partner belonging to the mono- versus co-infected group.

### Statistical analysis

This review aims to gather all available evidence regarding the effect of STI on HIV infectiousness, whether this was the primary objective of a study or not. Hence, heterogeneity in study design is inevitable. For example, HIV viral load can be measured at different anatomical sites, with different sampling techniques, for patients with different STI co-infections. Estimating a single summary statistic for such heterogeneous effects is challenging. Adopting a classical approach to conduct the meta-analysis would make it difficult to fit all the studies into one modelling framework. For example it might be necessary to choose a threshold and dichotomize data from studies providing continuous HIV viral load in order to compare them with the ones providing dichotomous data only. Hierarchical Bayesian models offer a flexible framework to coherently incorporate heterogeneous variables that theoretically relate to a common effect while providing estimates of the variability at each conceptual level [25].

We therefore used a Bayesian hierarchical model to estimate—across heterogeneous studies—an overall effect of STI co-infection on HIV viral load, while also estimating how this effect differed depending on the anatomical site sampled for viral load measurements and on the specific STI co-infection. Similarly, we also estimated how the effect of STI co-infection differed between studies and included individual-level random effects for longitudinal studies. Studies where the only outcome available was dichotomous (HIV viral load above or below a stated threshold) [10, 12, 26] were combined with studies that provided continuous outcomes by introducing latent variables [27]. We used uninformative priors for all effect sizes and their variances [28]. The details of the model and our prior choices are provided in Additional file 5. The model was developed in R version 3.0.2 [29] with package RSTAN version 2.2.0 [30]. Code is available upon request.

## Results

### Systematic review

**Fig. 1:**
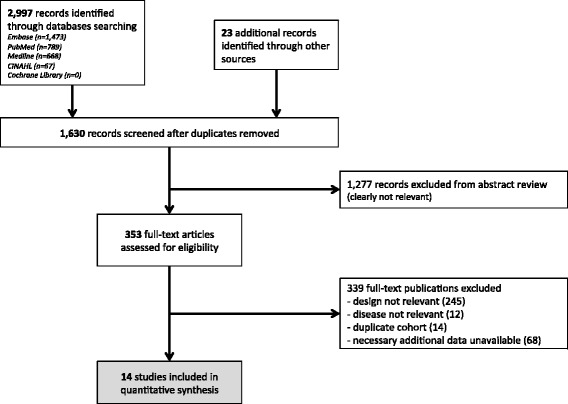
Flow chart of the selection process

Our database searches identified 2997 citations. In addition, 23 records were identified outside the database search (mostly via references cited in the publications retrieved from the database search). After duplicates were removed, 1630 records were assessed for eligibility (Fig. 1). We excluded 1277 records with abstracts that obviously did not meet our inclusion criteria, leaving 353 to be assessed with a full-text review. When studies appeared to have obtained but not published data relevant to our meta-analysis, we requested them from the original investigators. Of 73 investigators contacted for additional data, 21 responded and 10 provided data. This second screening identified 14 studies with sufficient information to be included in the meta-analysis [10, 12, 26, 31–41]. All studies included in our meta-analysis were approved by an ethics committee.

Table 1 summarizes the main characteristics of each eligible study. No studies involving discordant couples were eligible because none of them monitored HIV incidence and STI status at least monthly (our threshold to ascertain concomitance). Hence our findings are limited to an indirect measurement of HIV-infectiousness as measured by viral load at various anatomical sites. Of these 14 studies, two studies included participants from resource-limited countries [31, 34]; four studies focused on MSM [35–38]; and two reported on ART adherence [34, 40]. Syphilis, chlamydia and gonorrhea were the most reported STIs in the eligible studies. We provide further detail on the STI co-infections tested for and the anatomical sites sampled by each study in the Additional file 6.

Three studies included in this review [31, 33, 40] did not focus on differences in HIV infectiousness between STI co-infected and HIV mono-infected groups as a primary outcome. Among the 11 other studies, 7 suggest that co-infection with another genital infection may be associated with an increase in HIV viral load [10, 26, 35–39].

Table 2 outlines the main features from studies that could bias our estimates. The more STIs tested, the smaller the risk of incorrectly categorizing individuals as mono-infected. Six studies [32–34, 37, 39, 40] out of 14 reported on testing more than 4 STIs (in addition to HIV). Reporting adherence to ART reduces statistical misattribution of the reasons for increases in HIV viral load. Three studies [20, 34, 41] out of 14 explicitly reported adherence to ART. Because of HIV compartmentalization [42] (viral concentration may substantially differ between different anatomical sites within the same individual at a given time), measuring HIV viral load in genital or anal secretions is most relevant when considering sexual transmission. Eight out of 14 studies measured HIV viral load in such anatomical sites [10, 12, 26, 32, 34, 37–39].

### Meta-analysis

**Fig. 2:**
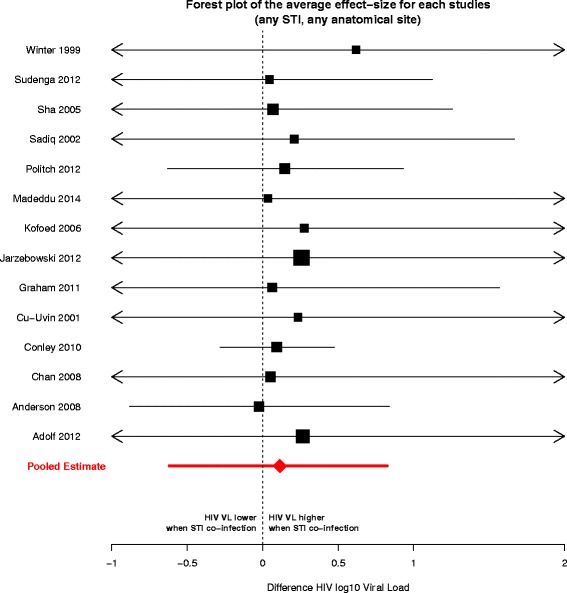
Posterior means and 95 % credible intervals of the effect size for each study included in the meta-analysis. The effect size is expressed as the difference of HIV viral load (log10) between an individual HIV positive, co-infected with any other STI and an individual only infected with HIV. The black square represents the posterior mean, with its area proportional to study sample size. The red diamond and arrows reflect estimates of the pooled effect (*i.e.*, across all studies, STIs and anatomical sites)

**Fig. 3:**
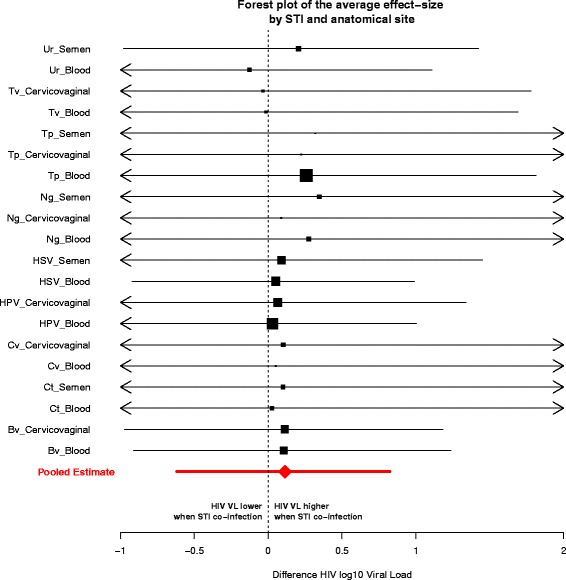
Forest plot for a given STI/anatomical site pair. Posterior means and 95 % credible intervals of the effect size for each study included in the meta-analysis. The effect size is expressed as the difference of HIV viral load (log10) between an individual HIV positive, co-infected with an STI and an individual only infected with HIV. The black square represents the mean of the distribution, its area is proportional to the number of data points associated with the specific STI/anatomical site pair; segments represent the 95 % credible intervals (CI). The red diamond shows the mean of the pooled effect (across all studies, STIs and anatomical sites) and the segment its 95 % CI. Bv: Bacterial vaginosis; Ct: *Chlamydia trachomatis*; Cv: Candidal vaginitis; Bacterial vaginosis; HPV: human papillomavirus; HSV: human simplex virus type 2; Ng: *Neisseria gonorrhoeae*; Tp: *Treponema pallidum*; Tv: *Trichomonas vaginalis*; Ur: urethritis

The 14 studies included in the meta-analysis represent 4607 visits from 2835 unique individuals (Table 3). The posterior distributions of effect sizes for each study, averaged across the associated STIs and anatomical sites, are shown in Fig. 2. We estimated that, among HIV-infected individuals on ART, the presence of another STI co-infection was associated with an HIV viral load 0.11 log10 (95 % CI −0.62 to 0.83) higher than in HIV mono-infected individuals, averaging over both anatomical sites and different STIs. Thus, we did not find a statistically significant effect of STI co-infection on the viral load of HIV-infected individuals on ART. Similarly, we did not find any statistically significant effect of STI co-infections on viral load when examining the effect on viral load as measured at a particular anatomical site, or for a particular STI co-infection, although we note that most of the posterior distributions for the effects of co-infection on viral load had positive means (Fig. 3).

**Fig. 4:**
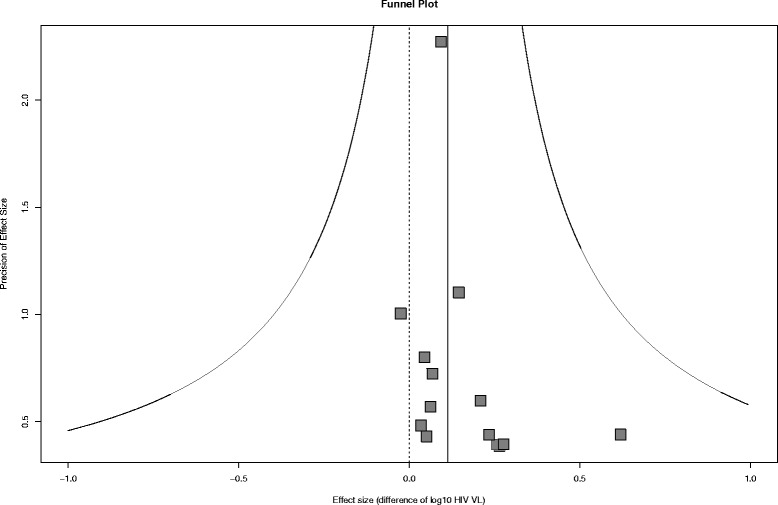
Funnel plot. The horizontal axis represents the mean posterior effect size for each study (the log10 HIV viral load between HIV positive individuals with and without STI co-infection), the vertical axis is the effect size precision (inverse of the standard deviation) of the associated study. Each point represents a study. The solid vertical line represents the mean effect size and the solid curved lines represent its 95 % CI. The dashed vertical line represents a null effect size (no HIV viral load difference)

Figure 4 shows a funnel plot of included studies and doesn’t exhibit a strong asymmetry, hence suggests no obvious publication bias. Another risk of bias across studies could have been introduced if temporal changes in STI prevalence and ART regimens were not adequately captured by the model.

The impact of heterogeneity between studies on the summary effect size (using the I^2^ statistic [43]) could not be reliably captured because of the large variance associated with our estimates (see Additional file 5). Three studies [31, 39, 40] exhibited HIV viral loads indicative of incomplete viral suppression in a non-negligible proportion of participants (*i.e.* HIV viral load above 4 log10). We did a sensitivity excluding these studies and did not find any qualitative differences in our results (see Additional file 7).

Visits where patients were co-infected with two or more STIs other than HIV (381 out of 4219 total visits) were not used in the main analysis because our model structure specified unique effects for each type of STI co-infection; we used a sensitivity analysis to explore the effects of this exclusion and found no qualitative impact on our results (see Additional file 7).

## Discussion

In the absence of qualifying studies that measured transmission risk directly, we conducted a meta-analysis of all available evidence of increased HIV viral load due to STI co-infection of individuals on ART, as a proxy for increased infection risk. Pooling information from all available studies, anatomical sites, and co-infections, we estimated that the average difference in viral load due to STI co-infection of individuals on ART was 0.11 log10 (95 % CI −0.62 to 0.83) greater than HIV mono-infected individuals.

Although our study provides some evidence for a small effect of STI co-infection on viral loads, we cannot rule out the possibility of no effect, or the possibility of a moderately large effect (the upper credible interval is 0.83 log10). Importantly, we are also not able to rule out the possibility that certain STIs (or certain combinations of STIs and anatomical site) have a much larger effect (see Fig. 3). Nonetheless, based on our analysis, we cautiously posit that ART manages—on average—to sustain its effectiveness at keeping HIV viral loads low during STI co-infection episodes, at the anatomical sites considered in this review (blood plasma, semen and cervicovaginal), and thus would be expected to maintain its effectiveness at preventing transmission.

Even with nearly 5000 data points used in this meta-analysis, realistic consideration of the variation between studies, STIs and anatomical sites reduces the statistical power considerably. There are other limitations that need to be highlighted.

Because eligible studies only estimated HIV viral loads, not transmission rates in discordant couples, our summary effect size is a proxy for HIV infectiousness and might not be an accurate representation of the actual sexual transmission risk. There were no eligible studies measuring rectal HIV viral load, so our estimate may not be applicable in assessing change in HIV infectiousness from receptive anal intercourse.

Not all studies tested for all STIs, hence we may have misclassified some co-infected individuals. This misclassification is likely, given the high prevalence rate of other STIs in the HIV-infected population [17]. Such misclassification could bias our estimates of the effect of STI on HIV viral load downwards.

Our study focused on whether STIs could interfere with viral suppression, and we therefore excluded HIV VL measurement done within 30 days of ART initiation. It seems likely that the effects of STIs on HIV viral load early in treatment are comparable to the effects before treatment begins and decline gradually through time, in an STI-specific fashion. We did not find any studies that could provide evidence bearing directly on the effect of STIs when ART has been initiated recently.

Adherence to ART was rarely reported or measured in the studies we used. Since the effect of STI co-infection on viral load would be stronger in people with poorly controlled viral loads, this effect is likely to bias our result. This bias will be exacerbated if co-infected individuals exhibit lower adherence (or could be reduced, or even reversed, if they are more likely to adhere, perhaps due to symptoms from the other STIs).

Most HIV viral load observations included in this meta-analysis were measured in blood plasma, not in genital secretions, which may limit the interpretation of our effect size to actual sexual transmission risk. Indeed, while plasma and genital HIV viral loads are correlated [44], evidences of HIV compartmentalization in some treated patients where viral loads as measured in genital secretions remain compatible with a non-negligible risk of transmission despite very low blood plasma viral loads [37, 45, 46].

We did not estimate gender-specific effects to avoid further complicating our model. It is possible that gender confounds our results to some extent. We note, however, that such confounding would be limited to blood measurements, since gender is implicitly accounted for in the other anatomical sites studied. Also, our estimate did not include a potential effect of menstrual cycle on genital HIV viral load, but we note that a recent study did not observe such effect [47].

If STIs were indeed an escape route for treatment as prevention, one could argue this should have shown up during large trials studying transmission rate among HIV discordant couples (for example [5]). But such trials do not provide complete reassurance, since trial participants are given STI monitoring and treatment not available to the general population.

The number of HIV-infected individuals receiving ART has dramatically increased during the last 10 years—from less than half a million in 2003 to about 13 million in 2013—and will increase further [18, 48]. The extended life expectancy associated with ART and the potentially higher exposure to STIs of the HIV-infected population may increase the prevalence of STI co-infections, particularly with public awareness of the decreased infectiousness of HIV while on ART and a potentially consequential decrease in condom usage. Hence, understanding the effects of such co-infections on HIV sexual transmission is an important public health issue.

Our study provides some insights into whether STI co-infections can undercut treatment as prevention efforts. Pooling available data, we estimate the degree to which STI co-infection may increase or decrease HIV shedding among treated individuals. We found a 95 % upper bound corresponding to a 0.83 log10 increase, which suggests that elevation of viral load by STI co-infections is unlikely to have a major impact on the ability of ART to reduce of HIV sexual transmission from patients on effective ART (as opposed to what could be observed in populations not on ART).

It is important to note that our results do not undercut the importance of control and treatment of STIs, not only for the well-being of infected individuals, but also for reducing HIV sexual transmission at the whole population level, which still has a majority of HIV infected individuals not on effective ART [18].

We did not have sufficient data to single out the effect of a specific STI on HIV viral load at a given anatomical site. Given the heterogeneous effect of STIs on HIV infectiousness, our analysis may have failed to identify epidemiologically relevant effects of particular STIs.

Hence, an important finding of our systematic review is that there is a paucity of available data with a sufficient level of detail to ascertain the effects of STI co-infection on the risk of HIV sexual transmission risk for individuals on ART. Future studies considering either transmission rate or HIV viral load may wish to consider the following suggestions: (i) given the possible high prevalence of co-infections among HIV-infected individuals, a broadest spectrum of relevant STIs should be tested; for transmission studies, testing should be performed frequently in both partners; (ii) HIV viral load should be measured in genital/rectal secretions, not blood plasma only; (iii) ART regimens and adherence should be reported, ideally at the patient level. We recognize that these suggestions may not always be practical, but when they can be followed they will help clarify the potentially important effects of STI-coinfection on the risk of HIV sexual transmission.

## Conclusions

Our findings suggest that, on average, ART maintains its effectiveness at controlling HIV viral load during STI co-infections. However, with currently available data, we cannot rule out the possibility that certain STI co-infections have a larger effect. More high-quality studies specifically aimed at investigating the impact of STI co-infection on HIV sexual transmission from individuals on ART are needed.

## Additional files

Additional file 1:
**PRISMA check list.**
Additional file 2:
**A protocol was prospectively registered in the PROSPERO database and is available at the following link:** http://www.crd.york.ac.uk/PROSPERO/display_record.asp?ID=CRD42013006056.
Additional file 3:
**STI positivity definitions.**
Additional file 4:
**Searches strategies.**
Additional file 5:
**Statistical model.**
Additional file 6:
**Figures summarizing meta-analysis data.**
Additional file 7:
**Sensitivity analysis.**
