# Supplementary material for: The effect of sexually transmitted co-infections on HIV viral load amongst individuals on antiretroviral therapy: a systematic review and meta-analysis
Source: BMC Infect Dis. 2015 Jun 30;15:249. doi: 10.1186/s12879-015-0961-5 (PMC4486691; doi:10.1186/s12879-015-0961-5)
Supplement: Additional file 2: — A protocol was prospectively registered in the PROSPERO database and is available at the following link: http://www.crd.york.ac.uk/PROSPERO/display_record.asp?ID=CRD42013006056. [file 12879_2015_961_MOESM2_ESM.docx]

A protocol was prospectively registered in the PROSPERO database and is available at the following link:

http://www.crd.york.ac.uk/PROSPERO/display_record.asp?ID=CRD42013006056
