# Supplementary material for: The effect of sexually transmitted co-infections on HIV viral load amongst individuals on antiretroviral therapy: a systematic review and meta-analysis
Source: BMC Infect Dis. 2015 Jun 30;15:249. doi: 10.1186/s12879-015-0961-5 (PMC4486691; doi:10.1186/s12879-015-0961-5)
Supplement: Additional file 3: — STI positivity definitions. [file 12879_2015_961_MOESM3_ESM.docx]

# Definition of STI positivity

An individual testing positive to any of the criteria below is categorized as infected with the associated STI.

**Chlamydia (*C. trachomatis*):** NAAT or culture or antigen detection (EIA)

**Gonorrhea (*N. gonorrhoeae*):** NAAT or culture

**Chancroids (*H. ducreyi*):** NAAT or culture

**Trichomoniasis (*T. vaginalis*):** culture or wet mount or NAAT

**Syphilis (*T.pallidum*):** dark field or VDLR or RPR or TPHA or FTA

That means we include not only ‘active’ stages (primary and secondary) but may also include a latent stage or even patients cured from syphilis (has we would not have any restriction on the nontreponemal titer)

**Human Papilloma Virus:** NAAT

**Herpes Simplex Virus 2:** serology or NAAT or culture

**Non-STI**

A woman testing positive to any of the criteria below is categorized as being positive with the associated infection:

**Bacterial vaginosis:** Amsel criteria or Nugent score>6

**Candidal vaginitis:** wet mount or culture or antigen detection
