# Supplementary material for: The effect of sexually transmitted co-infections on HIV viral load amongst individuals on antiretroviral therapy: a systematic review and meta-analysis
Source: BMC Infect Dis. 2015 Jun 30;15:249. doi: 10.1186/s12879-015-0961-5 (PMC4486691; doi:10.1186/s12879-015-0961-5)
Supplement: Additional file 4: — Searches strategies. [file 12879_2015_961_MOESM4_ESM.docx]

SUPPLEMENT – SEARCH STRATEGIES

# Medline –OVID


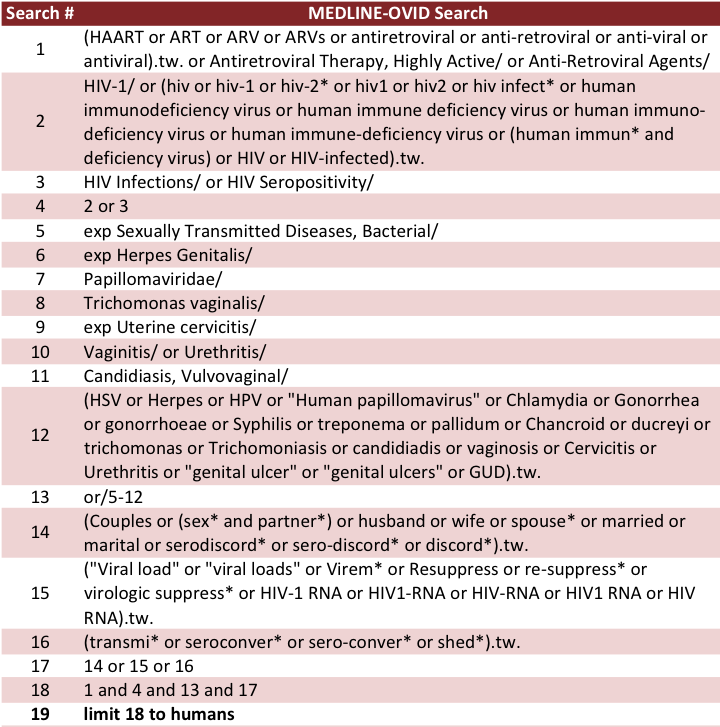


# Embase – OVID


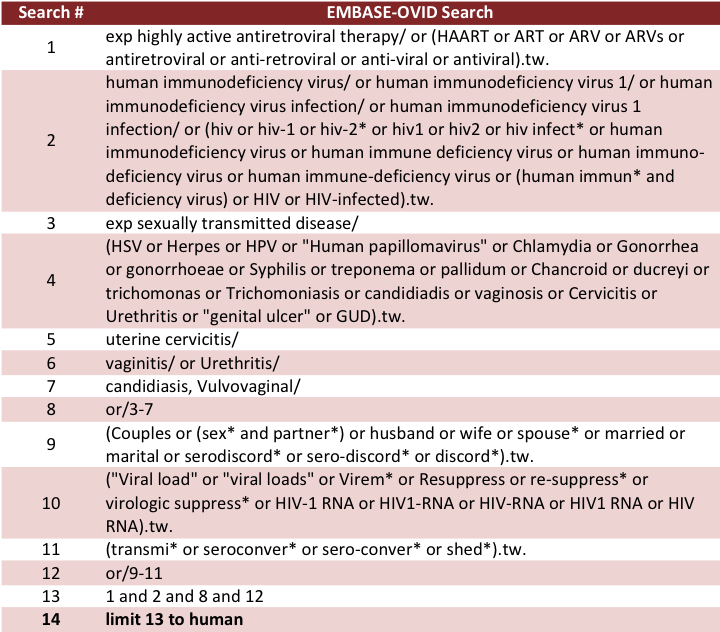


# CINAHL


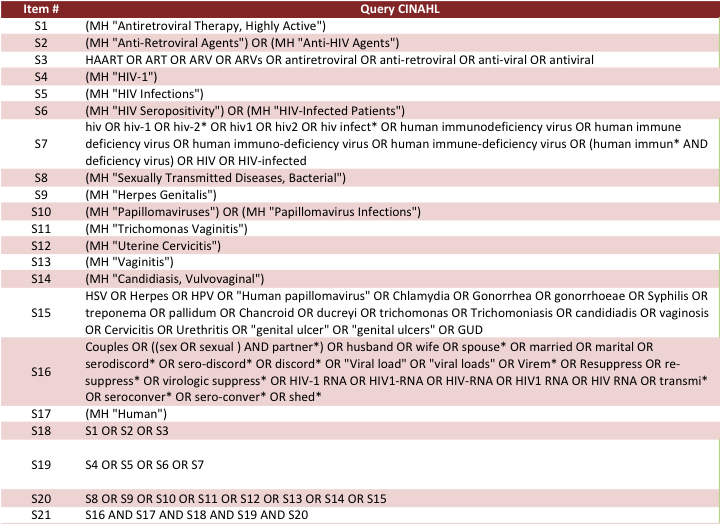


# Cochrane Library


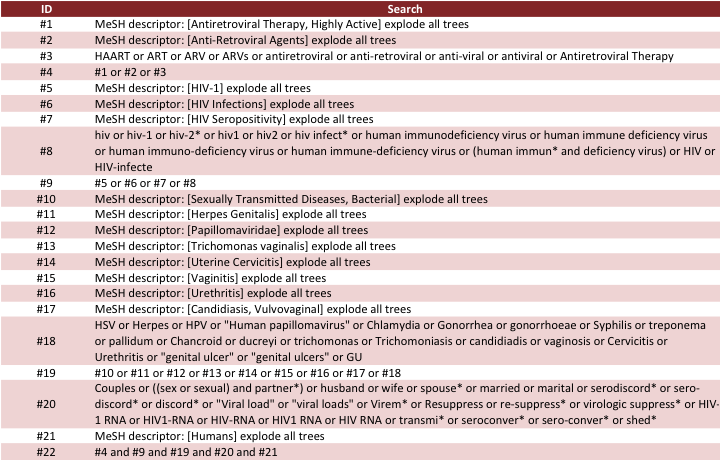


# PubMed

User Query:

((HAART[tw] OR ART[tw] OR ARV[tw] OR ARVs[tw] OR antiretroviral[tw] OR anti-retroviral[tw] OR anti-viral[tw] OR antiviral[tw] OR Antiretroviral Therapy, Highly Active[MeSH] OR Anti-Retroviral Agents[MeSH])

AND

(HIV-1[MeSH] OR hiv[tw] OR hiv-1[tw] OR hiv-2*[tw] OR hiv1[tw] OR hiv2[tw] OR hiv infect*[tw] OR human immunodeficiency virus[tw] OR human immune deficiency virus[tw] OR human immuno-deficiency virus[tw] OR human immune-deficiency virus[tw] OR (human immun*[tw] AND deficiency virus[tw]) OR HIV[tw] OR HIV-infected[tw] OR HIV Infections[MeSH] OR HIV Seropositivity[MeSH])

AND

(Sexually Transmitted Diseases, Bacterial[MeSH] OR Herpes Genitalis[MeSH] OR Papillomaviridae[MeSH] OR Trichomonas vaginalis[MeSH] OR Uterine cervicitis[MeSH] OR Vaginitis[MeSH] OR Urethritis[MeSH] OR Candidiasis, Vulvovaginal[MeSH] OR HSV[tw] OR Herpes[tw] OR HPV[tw] OR "Human papillomavirus"[tw] OR Chlamydia[tw] OR Gonorrhea[tw] OR gonorrhoeae[tw] OR Syphilis[tw] OR treponema[tw] OR pallidum[tw] OR Chancroid[tw] OR ducreyi[tw] OR trichomonas[tw] OR Trichomoniasis[tw] OR candidiadis[tw] OR vaginosis[tw] OR Cervicitis[tw] OR Urethritis[tw] OR "genital ulcer"[tw] OR "genital ulcers"[tw] OR GUD[tw])

AND

(Couples[tw] OR ((sex[tw] OR sexual[tw]) AND partner*[tw]) OR husband[tw] OR wife[tw] OR spouse*[tw] OR married[tw] OR marital[tw] OR serodiscord*[tw] OR sero-discord*[tw] OR discord*[tw] OR "Viral load"[tw] OR "viral loads"[tw] OR Virem*[tw] OR Resuppress[tw] OR re-suppress*[tw] OR virologic suppress*[tw] OR HIV-1 RNA[tw] OR HIV1-RNA[tw] OR HIV-RNA[tw] OR HIV1 RNA[tw] OR HIV RNA[tw] OR transmi*[tw] OR seroconver*[tw] OR sero-conver*[tw] OR shed*[tw])) AND

(Humans[Mesh])
