# Supplementary material for: The effect of sexually transmitted co-infections on HIV viral load amongst individuals on antiretroviral therapy: a systematic review and meta-analysis
Source: BMC Infect Dis. 2015 Jun 30;15:249. doi: 10.1186/s12879-015-0961-5 (PMC4486691; doi:10.1186/s12879-015-0961-5)
Supplement: Additional file 5: — Statistical model. [file 12879_2015_961_MOESM5_ESM.pdf]

Supplement: Modeling notes on The Effect of Sexually Transmitted co-Infections on HIV Infectiousness among Individuals on Antiretroviral Therapy: A Systematic Review and Meta-Analysis

David Champredon, Jonathan Dushoff

September 24, 2014

Contents

1 Background **2**

2 Data from studies **2**
2.1 Continuous outcomes at the individual level 2
2.2 Dichotomous outcomes 3

3 Model **3**
3.1 Universal Baseline HIV Viral Load 3
3.2 Hierarchy without co-infection 3
3.3 Hierarchy with co-infection 4
3.4 Dichotomous observations 5
3.5 Effect size variable 5

4 Implementation check **7**

5 Heterogeneity statistic **9**
5.1 Definition 9
5.2 Bayesian framework 9
5.3 Sensitivities of I^2 9

1 Background

This document describes the statistical framework of the systematic review and meta-analysis conducted in [1]. Briefly, our aim is to compare HIV viral loads between two groups:

- **HIV only group:** individuals under HIV antiretroviral therapy (ART) who are *not* co-infected with another sexually transmitted infection (STI)
- **Co-infected group:** individuals under HIV therapy who are co-infected with another STI

We want to assess, based on data collected from the systematic review, if there is an HIV viral load increase, at any given anatomical site, when an HIV-positive individual on ART is co-infected with another given STI.

2 Data from studies

Comparing HIV infectivity between the two groups can be done by either observing the number of (linked) transmissions within discordant couples or use the HIV viral load (preferably in genital secretion because we consider only sexual transmission here) as a proxy for infectiousness.

The systematic review retrieved different type of studies and each study has a particular data set. All eligible articles measured HIV viral load and none were transmissions studies.

HIV viral loads assays can measure different kind of viral signature: RNA cell-associated, RNA cell-free or DNA. Our model does not distinguish between RNA ca, RNA cf or DNA. However, this constraint should have a limited effect because we do have a study-level effect in our model (see Model section below). Most of the studies measured RNA levels (see Table 1 of main text [1]).

The common unit we use for all HIV viral loads is copies per millilitre (copies/mL).

Assays have detection limit which varies across studies. The range is from the order of 10 copies/mL to the order of 1000 copies/mL. (It is even possible to have different detection limit *within* a study because different assays were used for different anatomical sites)

Finally, HIV viral load was measured across different anatomical sites: blood plasma and genital secretions (semen for males, vagina or cervix for females). See Table 1 of main text for a summary.

2.1 Continuous outcomes at the individual level

Eligible studies where the HIV viral load is given for every participant are labeled "continuous" studies. We can have both a cross-sectional (one observation per individual) or longitudinal (multiple observations per individuals, usually through regular clinic visits) designs. Moreover, at a given visit, HIV viral load may have been measured from several anatomical sites.

2.2 Dichotomous outcomes

The systematic review also retrieved studies where only dichotomous outcomes have been reported. Typically, the dichotomous outcome is the HIV viral load being above an predefined threshold. The threshold is either chosen arbitrarily by the investigators or is implied by the detection limit of the assay. Hence, the data is represented with a 2 by 2 table counting the individuals, in the HIV only group and the co-infected one, having a viral load above or below a predetermined threshold.

3 Model

In order to have the best representation of the various random effects involved across all eligible studies retrieved from the systematic review, we choose a Bayesian hierarchical model as the framework for our meta-analysis[9].

Our data is represented by a HIV viral load as a function of the study s, the individual i of this study, her/his v^{th} visit (for longitudinal studies), the potential co-infecting STI d and the anatomical site a from where the HIV viral load was measured.

$$\text{Data Observed} = V[s,i,v,d,a] = \exp\big(VL[s,i,v,d,a]\big)$$

The viral load variable V is assumed to be on the *linear* scale, whereas VL is on the *natural log* scale[1]. This observed viral load is assumed to result from several effects, described below.

3.1 Universal Baseline HIV Viral Load

It is assumed that there is a 'universal' baseline viral load level B for individuals on ART and not co-infected with any other STI than HIV. The unit of universal baseline viral load is on the natural log scale and is assumed to vary according to the anatomical site from where it is measured, hence the notation B_A. We assume an uninformative uniform prior distributed between 0 and 20 ($e^{20} \simeq 5 \times 10^8$):

$$B_A[a] \sim U(0, 20)$$

for every anatomical site a.

3.2 Hierarchy without co-infection

From the universal level B_A for a given anatomical site, random effects for study, individual and visit levels are added in a hierarchical model.

For a given study s, the mean log HIV viral load is assumed to be normally distributed around the universal baseline value:

$$B_{\text{study}}[s,a] \sim \mathcal{N}(B_A[a]; \sigma_{B,\text{study}}[a])$$

[1]In the main text [1], parameters values reported were rescaled in the *log* base 10 as it is ubiquitous in the medical literature

Then, similarly, for the ith individual in this study, her/his baseline HIV viral load is distributed around the study-level value:

$$B_{\text{ind}}[s, i, a] \sim \mathcal{N}(B_{\text{study}}[s, a]; \sigma_{B,\text{ind}}[a])$$

The HIV viral load measured at the vth visit of individual i (if the study is not longitudinal, there is only one visit) is distributed around the value at the individual-level:

$$B_{\text{vis}}[s, i, v, a] \sim \mathcal{N}(B_{\text{ind}}[s, i, a]; \sigma_{B,\text{vis}}[a])$$

Hence, for an individual not co-infected with any other STI than HIV (coded as '$d = \emptyset$')

$$VL[s, i, v, \emptyset, a] = B_{\text{vis}}[s, i, v, a]$$

3.3 Hierarchy with co-infection

When the individual is co-infected with another STI d, the effect of this co-infection on the viral load is modelled by the following variables:

- $\alpha[a]$: change in baseline viral load at site a following co-infection with *any* STI
- $\delta[d]$: change in baseline viral load following co-infection with STI d, measured at *any* anatomical site
- $\gamma[d, a]$: change in baseline viral load following co-infection with STI, taking into account the correlation between the STI and its effect on a given anatomical site

Priors for these adjustments are again chosen in a uninformative way:

$$\begin{aligned}
\alpha[a] &\sim \mathcal{N}(0, \sigma_\alpha[a]) \\
\delta[d] &\sim \mathcal{N}(\bar{\delta}, \sigma_\delta[d]) \\
\gamma[d, a] &\sim \mathcal{N}(0, \sigma_\gamma[d, a])
\end{aligned}$$

The variable $\bar{\delta}$ represents the average effect of STI co-infection on HIV VL for an individual on ART, irrespective of the anatomical considered. It is assumed its prior distribution is uninformative:

$$\bar{\delta} \sim U(-10; 10)$$

Priors for the variances are all set to the same uninformative truncated normal distribution [3]:

$$\sigma_\bullet \sim \mathcal{N}_{R^+}(0, 2)$$

Similarly, a hierarchical structure is introduced to account for variabilities at the study, individual and visit levels:

$$\begin{aligned}
\alpha_{\text{study}}[s, a] &\sim \mathcal{N}(\alpha[a]; \sigma_{\alpha,\text{study}}[a]) \\
\alpha_{\text{ind}}[s, i, a] &\sim \mathcal{N}(\alpha_{\text{study}}[s, a]; \sigma_{\alpha,\text{ind}}[a])
\end{aligned}$$

$$\begin{aligned}\delta_{\text{study}}[s,d] &\sim \mathcal{N}(\delta[d];\sigma_{\delta,\text{study}}[d])\\ \delta_{\text{ind}}[s,i,d] &\sim \mathcal{N}(\delta_{\text{study}}[s,d];\sigma_{\delta,\text{ind}}[d])\end{aligned}$$

$$\begin{aligned}\gamma_{\text{study}}[s,d,a] &\sim \mathcal{N}(\gamma[d,a];\sigma_{\gamma,\text{study}}[d,a])\\ \gamma_{\text{ind}}[s,i,d,a] &\sim \mathcal{N}(\gamma_{\text{study}}[s,d,a];\sigma_{\gamma,\text{ind}}[d,a])\end{aligned}$$

Hence, for an individual co-infected with STI d, the log HIV viral load measured at anatomical site a is defined as:

$$\begin{aligned}VL[s,i,v,d,a] &= B_{\text{vis}}[s,i,v,a]\\ &\quad+\alpha_{\text{vis}}[s,i,v,a]\\ &\quad+\delta_{\text{vis}}[s,i,v,d]\\ &\quad+\gamma_{\text{vis}}[s,i,v,d,a]\end{aligned}$$

All variables being normally distributed, we have

$$VL[s,i,v,d,a] \sim \mathcal{N}(\Omega,\eta)$$

with

$$\Omega = \Omega[s,i,v,d,a] \;=\; B_{\text{ind}}[s,i,a] + \alpha_{\text{ind}}[s,i,a] + \delta_{\text{ind}}[s,i,d] + \gamma_{\text{ind}}[s,i,d,a]$$

$$\eta^2 = \eta^2[d,a] \;=\; \sigma^2_{B,\text{vis}}[a] + \sigma^2_{\alpha,\text{vis}}[a] + \sigma^2_{\delta,\text{vis}}[d] + \sigma^2_{\gamma,\text{vis}}[d,a]$$

3.4 Dichotomous observations

If the observation is dichotomous with threshold τ, the binary variable $D[s,i,v,d,a]$ is valued at 1 when the HIV viral load is above the threshold, 0 otherwise. We assume a Bernoulli distribution for the dichotomous variable D:

$$D[s,i,v,d,a] \sim \text{Bernoulli}\left(\ell\left(\frac{VL[s,i,v,d,a]-\tau}{\epsilon}\right)\right)$$

with $\ell(x) = 1/(1+e^{-x})$ and ϵ a tiny number (set at 0.01). In the dichotomous observation case, VL is not observed, but is a latent variable [2].

The full hierarchical structure of the model is illustrated in Figure 1.

3.5 Effect size variable

In order to have an easily interpretable effect-size, we define

$$ES[d,a] = \exp(\alpha[a]\,\delta[d]\,\gamma[d,a])$$

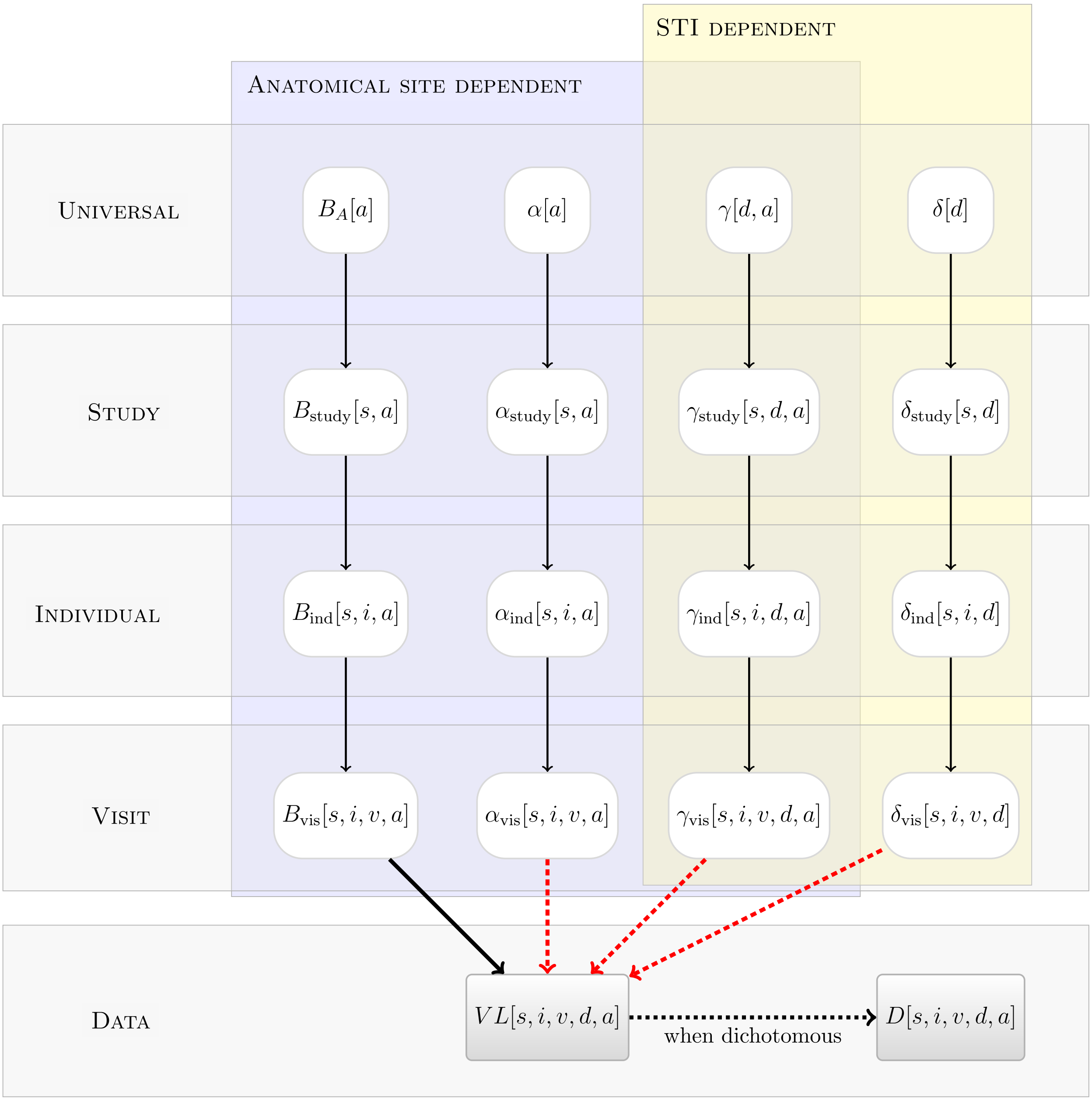

Figure 1: **Hierarchical model**. The horizontal shaded rectangles represent the various hierarchical levels: at the top the "universal" level, followed by finer levels i.e. study-,individual and visit-levels. The last rectangle represents the data used for Bayesian inference. The two vertical rectangles gather parameters that depend on anatomical sites and/or STIs. The solid black arrows show the hierarchical structure between parameters (see main text). The red dashed arrows illustrate Bayesian inference only when the data is associated with a STI co-infection. The black dashed arrow depicts the use of latent variables when the data is dichotomous. Notations are the same as in the main text.

The HIV log viral load at the highest hierarchical level, in the presence of co-infection d and measured at anatomical site a is given by

$$VL[d,a] = B_A[a] + \alpha[a] + \delta[d] + \gamma[d,a]$$

Expressing the viral load on the linear scale:

$$V[d,a] = e^{B_A[a]}\, ES[d,a]$$

Hence, $ES[d,a]$ is the multiplicative factor affecting the (linear) baseline HIV viral load at anatomical site a when co-infected with STI d.

When $ES[d,a] = 1$, no change in HIV viral load is expected; $ES[d,a] = 2$ means the sexually transmitted co-infection d is expected to double HIV shedding at anatomical site a.

Another interpretation of the effect-size (widespread in the medical literature) is to consider the quantity $\log(ES)/\log(10)$ that represents the HIV viral load difference, in log10 copies/mL, between a typical HIV positive individual on ART who has another sexually transmitted co-infection and an individual who has not.

Effect size for a given study

HIV viral loads measured are structurally distinguished by anatomical site and co-infection. Hence, in this framework, the variable (named ES_{study}) that represents co-infection effect on HIV viral load of a given study must specify which anatomical site and STI are considered. So for study s we define such effect by:

$$ES_{\text{study}}[s] = \exp\big(\alpha[s,a]\,\delta[s,d]\,\gamma[s,d,a]\big)$$

It is also possible to evaluate, for a given study, the mean effect of STI co-infection on HIV viral load across all anatomical sites and infections considered in that study. Hence, we define such mean study effect by

$$ES_{\text{avg\_study}}[s] = \exp\left(\frac{1}{AD}\sum_{a,d=1}^{a=A,d=D}\alpha[s,a]\,\delta[s,d]\,\gamma[s,d,a]\right)$$

with A (resp. D) the number of anatomical sites (resp. STIs) considered in study s.

4 Implementation check

The model described above is implemented in R[7], using the package RStan[8] for sampling the Markov chains.

In order to test the implementation is free of errors that would affect parameter estimations, we generated simulated data from known parameter values and checked they are correctly estimated by the hierarchical model.

The simulated data were designed to be similar to the real data, that is a hierarchical structure universal/study/individuals/visits and mix of continuous and dichotomous studies, anatomical sites, STIs. The only major difference with the real data set is the simulated one is much smaller, in order to achieve a faster convergence and hence a more accurate estimation of the known parameters. Because the aim is to check the implementation, working on a smaller data set is not an issue. The simulated data set was created with 3 continuous studies, 2 dichotomous ones; each study had a maximum of 35 individuals; each individual had 2 visits. These studies were simulated with 3 STIs and 3 anatomical sites. The STI prevalence was 0.33 for all studies.

The estimation of universal-level parameters B_A, α, δ and γ is shown in Figure 2 and confirms the implementation is likely free of major errors impacting estimation of parameters.

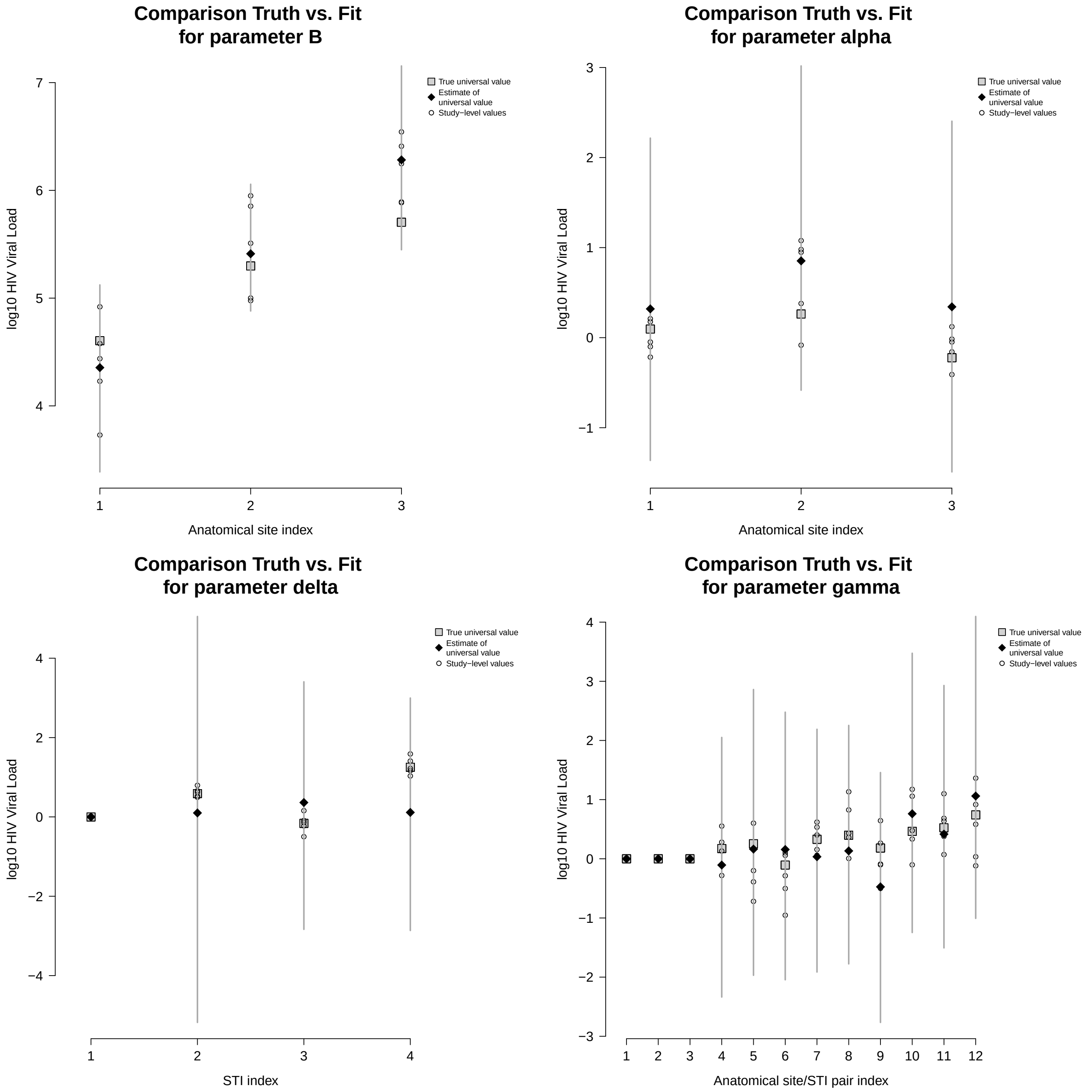

Figure 2: **Model sanity check.** Estimation of universal-level parameters from simulated data.The shaded square represents the 'true' level of the universal-level parameter. The filled diamond is the estimated parameter mean and the vertical segment represents the 95% credible interval. The open circles represent the study-level parameters (this is for information only: the study-level values may be sampled relatively far from the universal mean, affecting the estimation of the universal level value). This fit was done with 3 chains, each having 30,000 iterations (including half for warm-up). Markov chains Monte-Carlo convergence was confirmed by requiring $\hat{R} < 1.02$ for all parameters[4]. STI index 1 represents no co-infection, hence we always have $\delta[1] = 1$ and $\gamma[1, a] = 1$ (for any anatomical site a); this explains why there is no estimation error for the first point of δ and the first 3 points for γ. 'True' values (shaded squares) are all within the credible intervals and the mean is most of the time very close to this 'true' value, comforting an implementation likely free of major errors.

5 Heterogeneity statistic

In order to assess the effect of heterogeneity between studies, we use the I^2 statistic as it is easily interpretable and ubiquitous in meta-analysis[5].

5.1 Definition

The statistic I^2 is defined as [6]:

$$I^2 = \frac{Q - (n-1)}{Q}$$

with Q the Cochrane's (1954) heterogeneity statistic and n the total number of studies.

In a frequentist, fixed effect framework, we have

$$Q = \sum_{i=1}^{n} w_i (y_i - \mu)^2$$

with y_i the estimated effect-size for the ith study, $w_i = 1/\sigma_i^2$ the inverse of the of the estimated variance of the ith study and μ the summary estimate effect-size when pooling all studies together for the meta-analysis. Still in this framework (fixed-effect), we have $\mu = (\sum w_i y_i)/\sum w_i$.

If estimates y_i are assumed to be normally distributed around μ, then Q has a χ^2 distribution with $n-1$ degrees of freedom. Heterogeneity can then be tested, and is usually reported as a p-value $p(\chi^2 > Q)$ (a low p-value means significant heterogeneity).

Note that I^2 can be negative. In this case, it is reported as 0 ($I^2 = \max(I^2; 0)$)

But it has been reported that Q may mislead because of too little power when dealing with few studies and too much power when dealing with numerous and large studies[5]. A suggested alternative is to use I^2, especially to assess the *effect* of heterogeneity on the summary effect size.

5.2 Bayesian framework

In a Bayesian framework, the definition of Q and I^2 is the same. Only the weights w_i and μ require some clarification.

We take w_i as the inverse of the estimated variance of the posterior distribution of the effect size associated with the ith study; and μ is the mean of the posterior distribution of the summary effect size.

5.3 Sensitivities of I^2

For meta-analysis with large variances of the estimated effect sizes, both Q and I^2 tend to be smaller. Indeed – for the same mean value of estimates y_i – both Q and I^2 decrease as the estimated variances increase:

$$\frac{\partial Q}{\partial \sigma_i^2} = -\sum_i \frac{(y_i - \mu)^2}{\sigma_i^4} < 0$$

$$\frac{\partial I^2}{\partial \sigma_i^2} = \frac{n-1}{Q^2}\frac{\partial Q}{\partial \sigma_i^2} < 0$$

This make sense as I^2 describes the percentage of total variation across studies due to genuine heterogeneity, rather than chance[5]. The larger the variance, the more heterogeneity could be explained by chance alone.

References

[1] David Champredon, Steve E Bellan, Wim Delva, Spencer Hunt, Chyun-Fung Shi, Marek Smieja, and Jonathan Dushoff. The Effect of Sexually Transmitted co-Infections on HIV Infectiousness among Individuals on Antiretroviral Therapy: A Systematic Review and Meta-Analysis.

[2] Francesca Dominici and Giovanni Parmigiani. Combining Studies with Continuous and Dichotomous Responses: A Latent-Variables Approach . In Dalene K Stangl and Donald A Berry, editors, *Meta-Analysis in Medicine and Health Policy.* Marcel Dekker, New-York, January 2000.

[3] Andrew Gelman. Prior distributions for variance parameters in hierarchical models. *Bayesian analysis*, 1(3):515–533, 2006.

[4] Andrew Gelman, John B Carlin, Hal S Stern, David B Dunson, Aki Vehtari, and Donald B Rubin. *Bayesian Data Analysis, Third Edition.* CRC Press, November 2013.

[5] Julian Higgins, Simon G Thompson, Jonathan J Deeks, and Douglas G Altman. Measuring inconsistency in meta-analyses. *BMJ*, 327(7414):557–560, 2003.

[6] Julian P T Higgins and Simon G Thompson. Quantifying heterogeneity in a meta-analysis. *Statistics in Medicine*, 21(11):1539–1558, 2002.

[7] R Core Team. *R: A Language and Environment for Statistical Computing.*

[8] Stan Development Team. *Stan: A C++ Library for Probability and Sampling,Version 2.2*, 2014.

[9] Alex J Sutton and Keith R Abrams. Bayesian methods in meta-analysis and evidence synthesis. *Statistical Methods in Medical Research*, 10(4):277–303, 2001.
