# Supplementary material for: The effect of sexually transmitted co-infections on HIV viral load amongst individuals on antiretroviral therapy: a systematic review and meta-analysis
Source: BMC Infect Dis. 2015 Jun 30;15:249. doi: 10.1186/s12879-015-0961-5 (PMC4486691; doi:10.1186/s12879-015-0961-5)
Supplement: Additional file 6: — Figures summarizing meta-analysis data. [file 12879_2015_961_MOESM6_ESM.pdf]

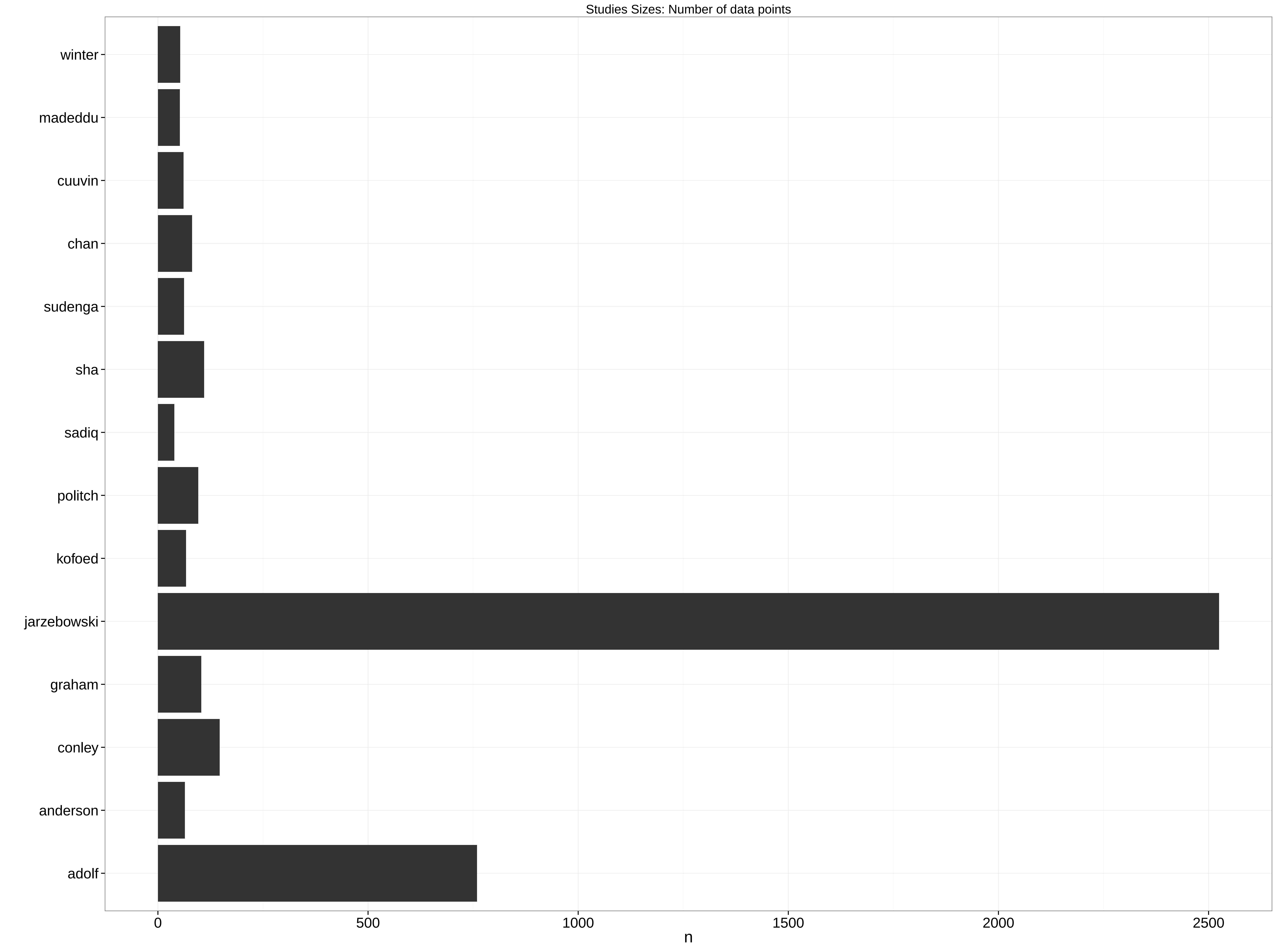
Studies Sizes: Number of data points
winter
madeddu
cuuvin
chan
sudenga
sha
sadiq
politch
kofoed
jarzebowski
graham
conley
anderson
adolf
0
500
1000
1500
2000
2500
n

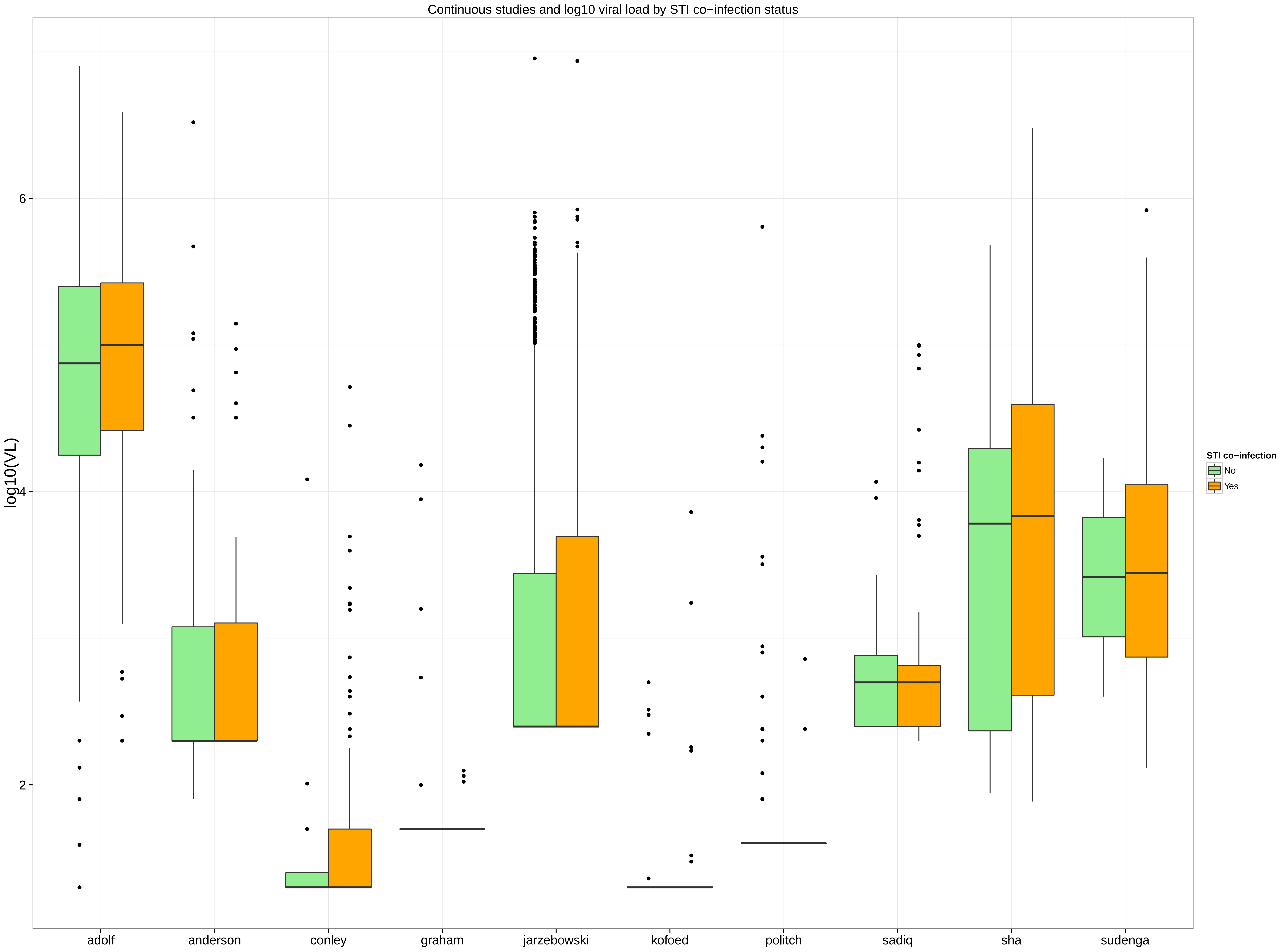
Continuous studies and log10 viral load by STI co−infection status
log10(VL)
6
4
2
adolf
anderson
conley
graham
jarzebowski
kofoed
politch
sadiq
sha
sudenga
STI co−infection
No
Yes

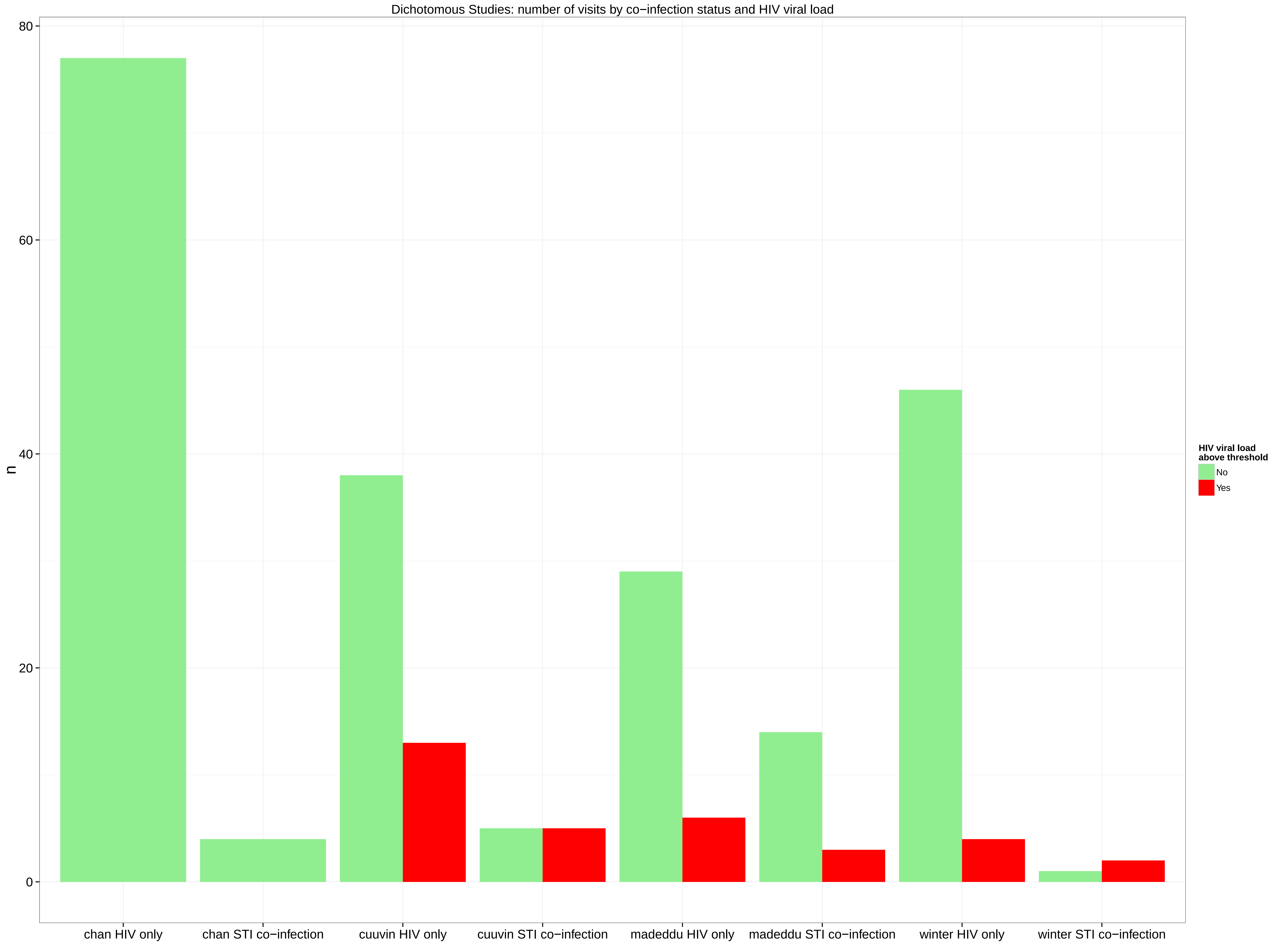
Dichotomous Studies: number of visits by co−infection status and HIV viral load
80
60
40
20
0
n
chan HIV only
chan STI co−infection
cuuvin HIV only
cuuvin STI co−infection
madeddu HIV only
madeddu STI co−infection
winter HIV only
winter STI co−infection
HIV viral load above threshold
No
Yes

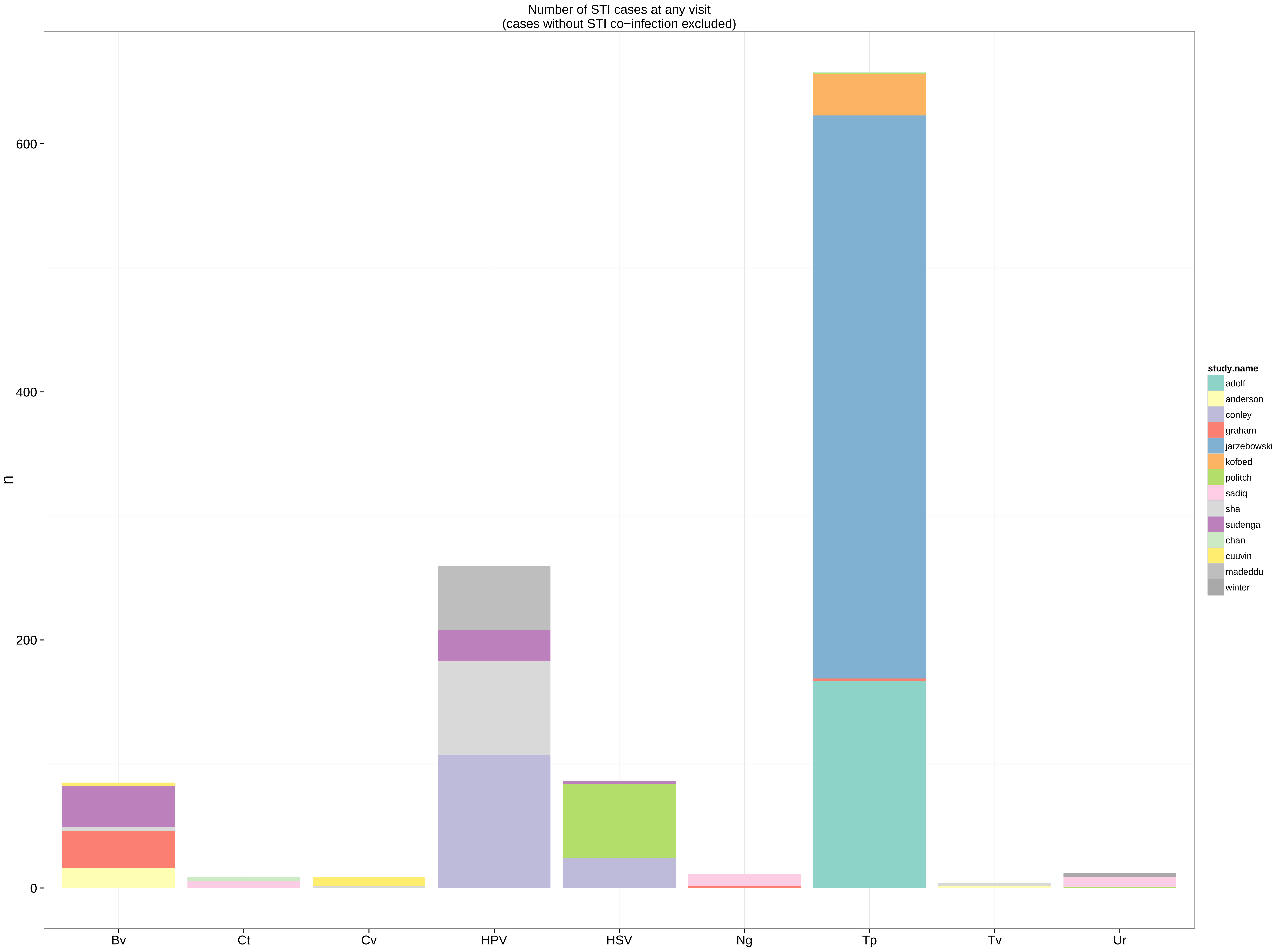
Number of STI cases at any visit
(cases without STI co−infection excluded)
n
600
400
200
0
Bv
Ct
Cv
HPV
HSV
Ng
Tp
Tv
Ur
study.name
adolf
anderson
conley
graham
jarzebowski
kofoed
politch
sadiq
sha
sudenga
chan
cuuvin
madeddu
winter

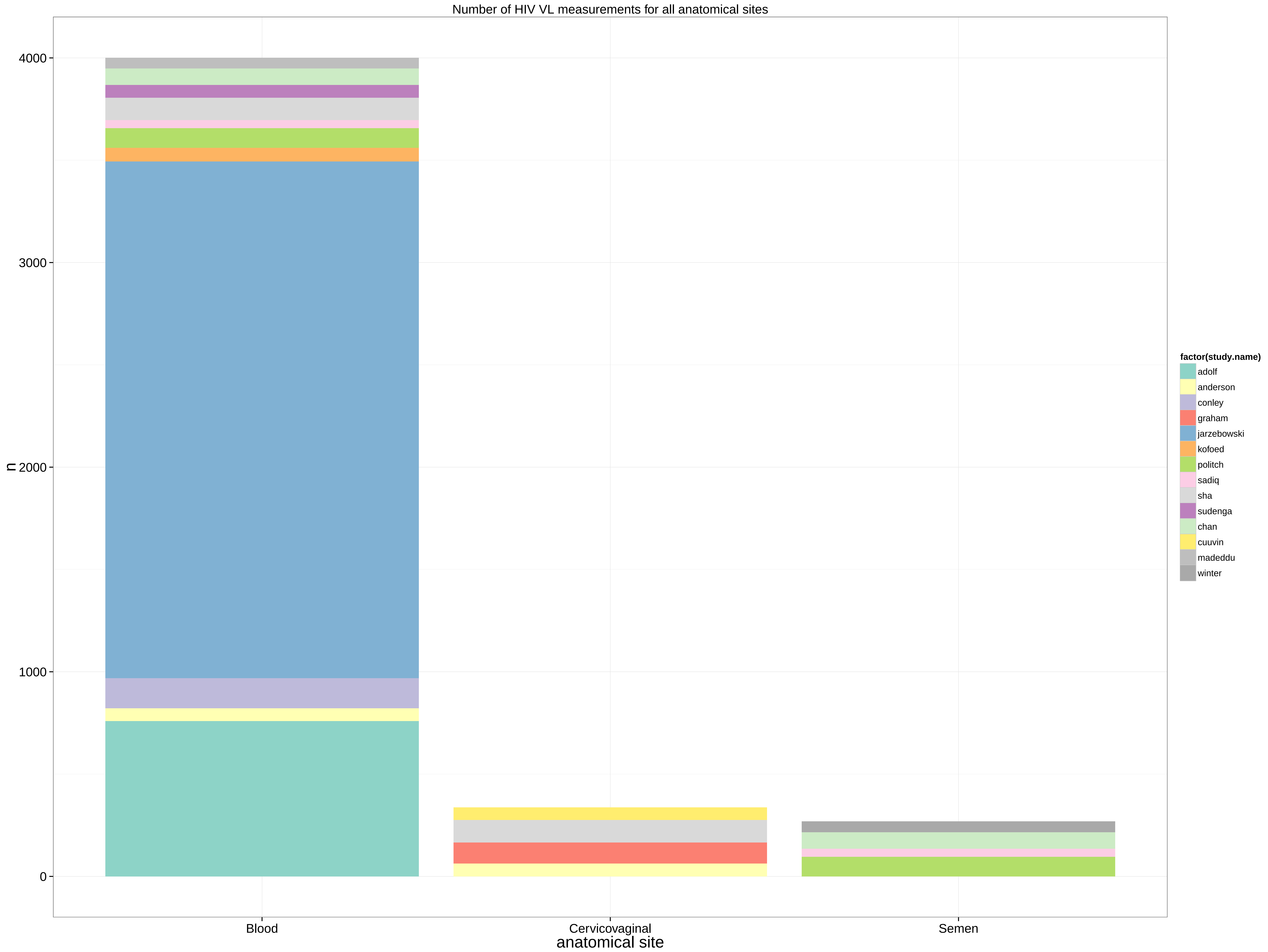
Number of HIV VL measurements for all anatomical sites
4000
3000
2000
1000
0
n
Blood
Cervicovaginal
Semen
anatomical site
factor(study.name)
adolf
anderson
conley
graham
jarzebowski
kofoed
politch
sadiq
sha
sudenga
chan
cuuvin
madeddu
winter

Gender and STI co−infection status

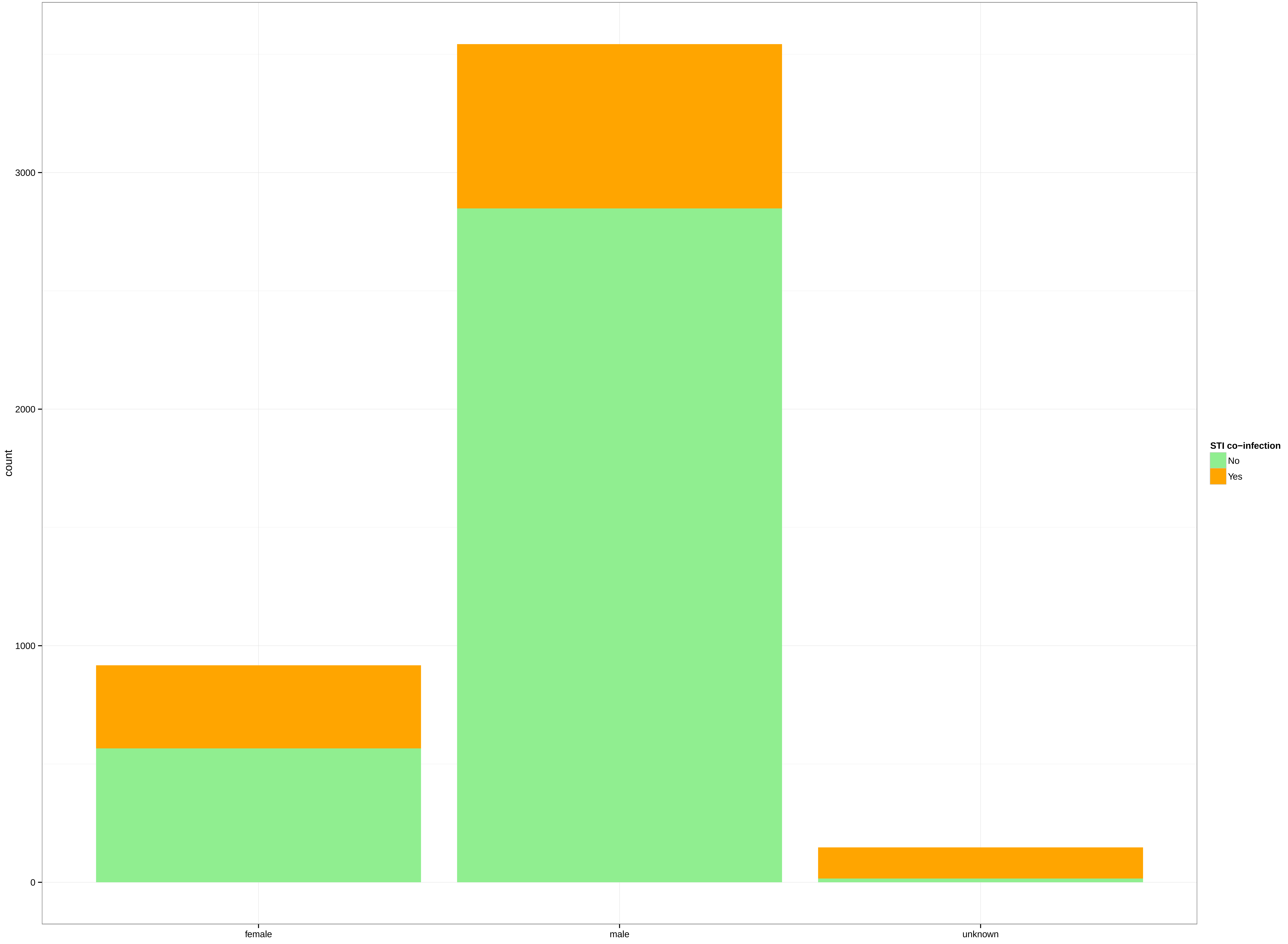

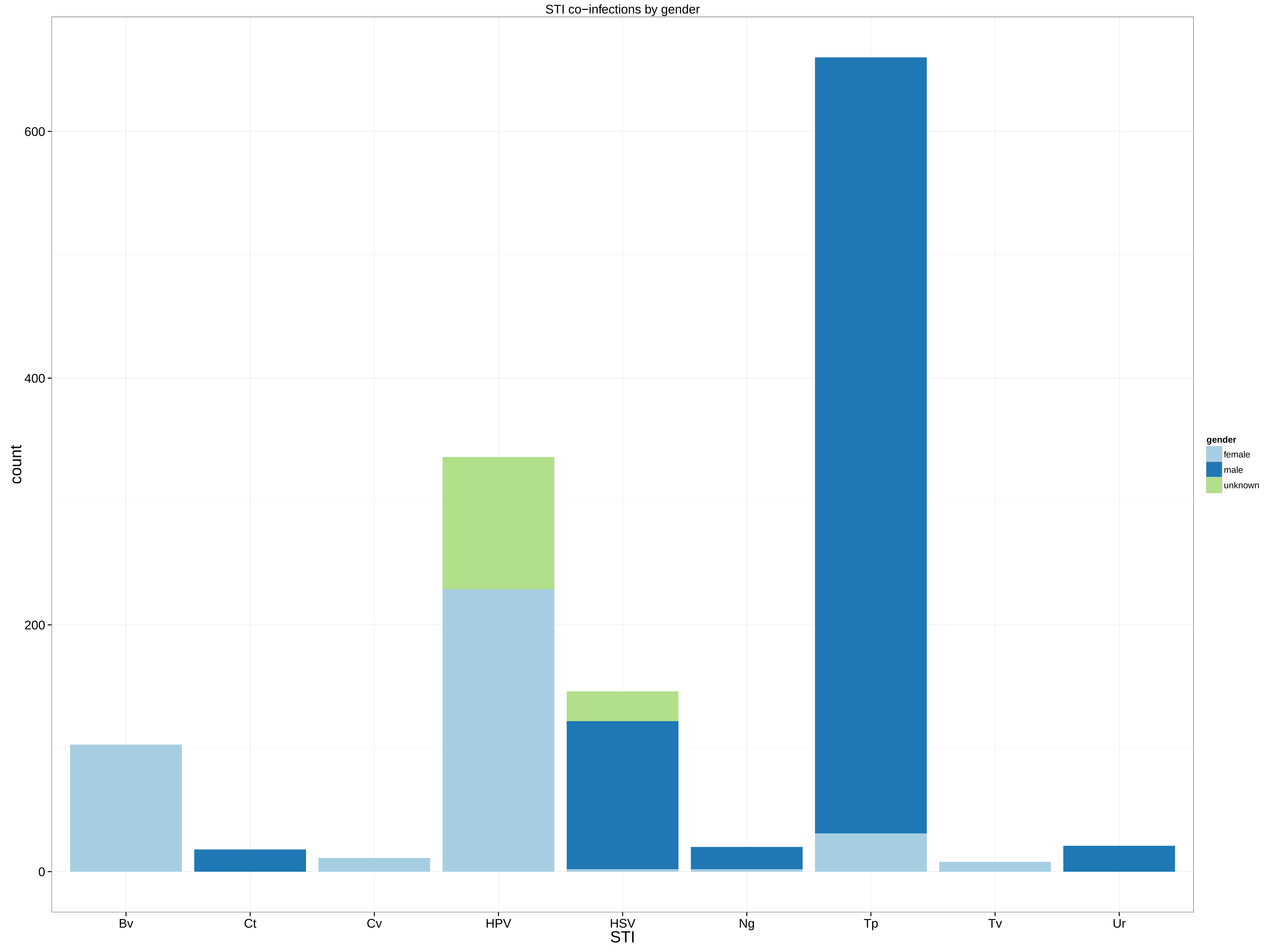

STI co−infections by gender
count
600
400
200
0
Bv
Ct
Cv
HPV
HSV
Ng
Tp
Tv
Ur
STI
gender
female
male
unknown

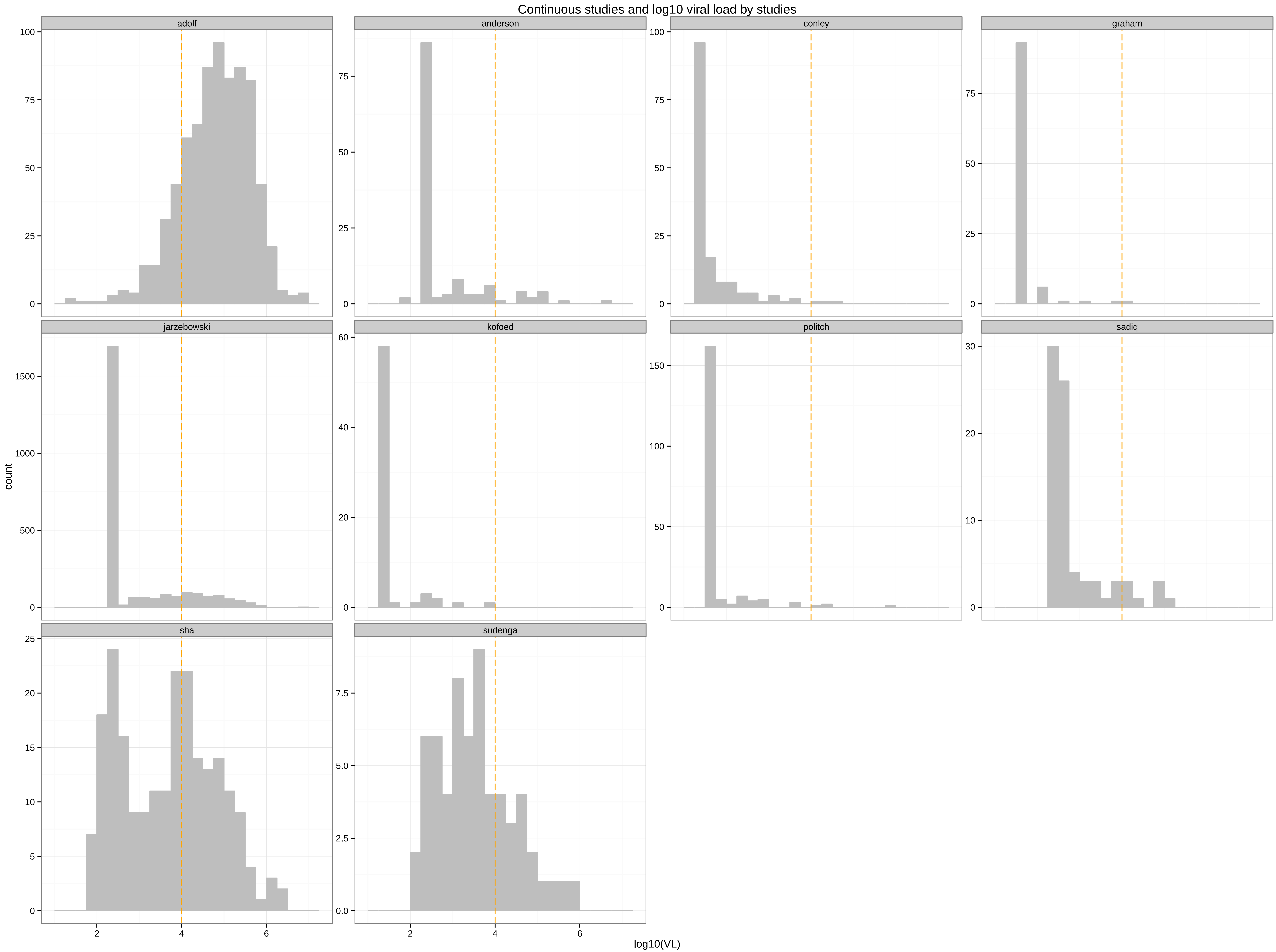

Continuous studies and log10 viral load by studies
adolf
anderson
conley
graham
jarzebowski
kofoed
politch
sadiq
sha
sudenga
count
log10(VL)

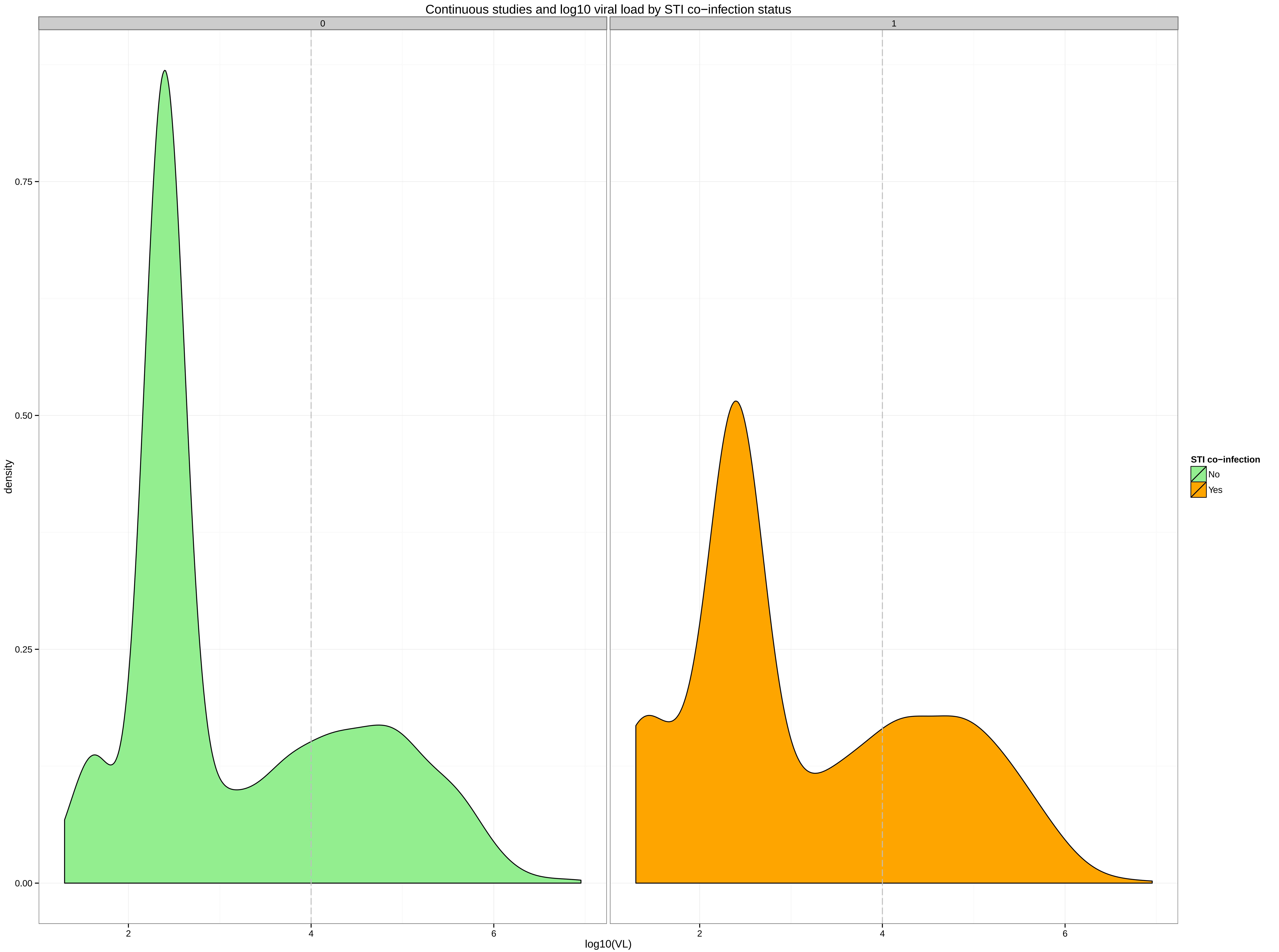
Continuous studies and log10 viral load by STI co−infection status
0
1
density
0.00
0.25
0.50
0.75
2
4
6
log10(VL)
STI co−infection
No
Yes
