# Supplementary material for: The effect of sexually transmitted co-infections on HIV viral load amongst individuals on antiretroviral therapy: a systematic review and meta-analysis
Source: BMC Infect Dis. 2015 Jun 30;15:249. doi: 10.1186/s12879-015-0961-5 (PMC4486691; doi:10.1186/s12879-015-0961-5)
Supplement: Additional file 7: — Sensitivity analysis. [file 12879_2015_961_MOESM7_ESM.docx]

Supplement – Sensitivity Analysis

# Figure A - Censoring multiple STI co-infections

To assess the impact of censoring multiple STI co-infections, several other estimations were performed using different data sets. Because our statistical model handles only one STI co-infection, we introduced the concept of “dominant STI”: out of n co-infecting STIs, one is the dominant infection in driving a potential response on HIV viral load.

The estimation of the effect size using the data set censoring multiple STI co-infections was compared with the estimation using the data set applying the dominant STI methodology, where several data sets were used: taking the first, second or third (if it existed) STI listed as the dominant one. All estimations were run with 2 chains with 35,000 iterations each.

# Figure B - Outlier studies

Three studies {Adolf:2011kn, Sha:2005tm, Sudenga:2012db} have a large proportion of high (>10^4^ copies/mL) HIV viral load among patients on ART. We looked at the impact of these three studies on the estimated pool effect size by removing them from the data set. The effect size when outlier studies are removed has a larger credible interval (less data points) and its level is not qualitatively different from the one estimated with full data set.

(Both estimations were run with 4 chains with 40,000 iterations each)
